# Sliding window haplotype approaches overcome single SNP analysis limitations in identifying genes for meat tenderness in Nelore cattle

**DOI:** 10.1186/s12863-019-0713-4

**Published:** 2019-01-14

**Authors:** Camila U. Braz, Jeremy F. Taylor, Tiago Bresolin, Rafael Espigolan, Fabieli L. B. Feitosa, Roberto Carvalheiro, Fernando Baldi, Lucia G. de Albuquerque, Henrique N. de Oliveira

**Affiliations:** 1 Animal Science Department, São Paulo State University (Unesp), Jaboticabal, SP 144884-900 Brazil; 2 Division of Animal Sciences, University of Missouri, Columbia, MO 65211 USA

**Keywords:** Additive genetic variance, Beef cattle, Haplotype, GWAA, Meat tenderness

## Abstract

**Background:**

Traditional single nucleotide polymorphism (SNP) genome-wide association analysis (GWAA) can be inefficient because single SNPs provide limited genetic information about genomic regions. On the other hand, using haplotypes in the statistical analysis may increase the extent of linkage disequilibrium (LD) between haplotypes and causal variants and may also potentially capture epistastic interactions between variants within a haplotyped locus, providing an increase in the power and robustness of the association studies. We performed GWAA (413,355 SNP markers) using haplotypes based on variable-sized sliding windows and compared the results to a single-SNP GWAA using Warner-Bratzler shear force measured in the *longissimus thorasis* muscle of 3161 Nelore bulls to ascertain the optimal window size for identifying the genomic regions that influence meat tenderness.

**Results:**

The GWAA using single SNPs identified eight variants influencing meat tenderness on BTA 3, 4, 9, 10 and 11. However, thirty-three putative meat tenderness QTL were detected on BTA 1, 3, 4, 5, 8, 9, 10, 11, 15, 17, 18, 24, 25, 26 and 29 using variable-sized sliding haplotype windows. Analyses using sliding window haplotypes of 3, 5, 7, 9 and 11 SNPs identified 57, 61, 42, 39, and 21% of all thirty-three putative QTL regions, respectively; however, the analyses using the 3 and 5 SNP haplotypes, cumulatively detected 88% of the putative QTL. The genes associated with variation in meat tenderness participate in myogenesis, neurogenesis, lipid and fatty acid metabolism and skeletal muscle structure or composition processes.

**Conclusions:**

GWAA using haplotypes based on variable-sized sliding windows allowed the detection of more QTL than traditional single-SNP GWAA. Analyses using smaller haplotypes (3 and 5 SNPs) detected a higher proportion of the putative QTL.

**Electronic supplementary material:**

The online version of this article (10.1186/s12863-019-0713-4) contains supplementary material, which is available to authorized users.

## Background

Following the completion of the first draft bovine reference genome assembly, a high-density single nucleotide polymorphism (SNP) genotyping assay was developed [1], enabling genome-wide association analyses (GWAA), which are useful in understanding the underlying biology of traits [2, 3]. Several GWAA have identified SNP associated with meat tenderness in cattle [4–7], which is one of the most important attributes impacting consumer satisfaction and the price of beef [8]. Moreover, meat tenderness is the most important trait requiring improvement in Nelore cattle in order to increase beef quality and ensure consumer acceptability. However, studies have shown that the traditional single-SNP GWAA can be inefficient because single-SNPs provide limited information about the content of flanking genomic regions [9, 10]. On the other hand, using haplotypes in the statistical analysis may increase the extent of linkage disequilibrium (LD) between haplotypes and causal variants and also potentially capture epistastic interactions between variants within a haplotyped locus, providing increased power and robustness for association studies [10–16].

The use of haplotypes in GWAA analysis has not been widely exploited because there is no consensus on how many adjacent SNPs should be haplotyped, or on what is the best methodology for the definition and analysis of the haplotype blocks [17]. Different criteria have been proposed, but the most widely used approach is based on the extent of LD between markers as described by [18], which combines SNPs into haplotype blocks in genomic regions of high LD, i.e., with low evidence for recombination. However, the use of this approach can result in “orphan” SNPs that fall outside of predefined LD blocks, leading to a loss in ability to dissect genetic variation in these regions in the association analysis [14]. Thus, haplotype block approaches may not be the most efficient approach for association studies [19]. An alternative strategy for performing haplotype-based association analyses is based on overlapping sliding windows, in which several neighboring contiguous SNPs are included in a haplotype analysis, spanning the entire genome regardless of the extent of LD between the markers [10, 14, 20]. This approach has been shown to be more powerful than single-SNP and LD block–based haplotype analyses, particularly in genomic regions with high recombination and low LD [10, 21, 22]. Furthermore, according to [10], the use of sliding window haplotype analysis increases the likelihood of QTL detection and identification of the genomic regions harboring the causal variants.

We performed a GWAA using haplotypes defined by variable-sized sliding windows and compared the results to single-SNP GWAA to ascertain the optimal window size for identifying QTL regions that influence meat tenderness in Nelore cattle. The results of this study provide a better understanding of the genetic basis of meat tenderness in zebu cattle.

## Methods

### Animals

The 3161 Nelore bulls used in this study were born between 2008 and 2013 and were sourced from three different animal breeding programs. These animals were raised on pasture, finished in a feedlot for approximately 90 days, and then slaughtered in commercial slaughterhouses at a mean age of 691 ± 102 days. Contemporary groups (CG) were defined by the combination of farm of origin, year of birth, management group as long-yearlings and month and year of slaughter. The animals belonged to 116 CG with at least three animals per group.

### Phenotypic and genotypic data

The animals were slaughtered in commercial slaughter houses and the carcasses were identified by tags and chilled for 24 to 48 h *post-mortem*. Steaks of 2.54 cm thickness were then collected from the *longissimus thoracis* muscle between the 12th and 13th ribs from the left half of the carcasses. The steaks were vacuum sealed and aged in a cold chamber for 150 h at 1 °C and then were frozen at − 20 °C. Next, the steaks were cooked in an oven to an internal temperature of 71 °C as proposed by [23]. Warner-Bratzler shear force (WBSF), a mechanical measurement of tenderness, was measured 24 h after cooking using a Salter shearing device (G-R Electric, Manhattan, KS). Eight 1.27 mm meat cores were obtained from each sample and the average shear force of the eight cores was used in analysis. The mean WBSF for the 3161 Nelore bulls was 5.9 ± 1.80 kg varying from 1.6 kg to 11.9 kg.

Using a DNeasy Blood & Tissue Kit (Qiagen GmbH, Hilden, Germany), tissues from the *longissimus thoracis* muscle were used to extract DNA according to the manufacturer’s instructions. Genotyping was performed by high-density BeadArray technology (777 K) using the Illumina (San Diego, CA) BovineHD Infinium Assay® with an Illumina HiScan System® for 1405 animals and the 1756 remaining animals were genotyped with a lower density bead array (GGP75Ki). Genotypes were imputed to the content of the BovineHD assay and phased using FImpute software [24] including available pedigree information. The average imputation accuracy from GGP75Ki to Illumina® BovineHD was 98.93%, as reported by [25]. Samples for which the genotype call rate was less than 90% and SNP markers with a call rate of less than 95%, or that had a minor allele frequency of less than 5%, or Hardy Weinberg Equilibrium test statistic probability of less than 10^− 5^ or that were unmapped to autosomes or sex-linked were removed. A total of 413,355 SNP markers remained for analysis. The genomic coordinates for each SNP marker were based on the *Bos taurus* UMD3.1 reference assembly.

### Construction of haplotypes

Five different haplotype sizes were constructed based on overlapping sliding windows methodology spanning the entire genome: three (SW3), five (SW5), seven (SW7), nine (SW9) or eleven (SW11) SNPs. Given an ordered set of markers (SNP_1_, SNP_2_, SNP_3_, ..., SNP_*n*_), where *n* is the number of SNP markers on the chromosome, sliding windows of overlapping haplotypes are tested in sequence. i.e. for SW3: haplotype 1 (SNP_1_-SNP_2_-SNP_3_), haplotype 2 (SNP_2_-SNP_3_-SNP_4_), haplotype 3 (SNP_3_-SNP_4_-SNP_5_), ..., haplotype *n* (SNP_*n-2*_, SNP_*n-1*_, SNP_*n*_) [14, 20]. The haplotypes were estimated for each locus and the diplotype of each animal was estimated as the combination of haplotypes *i* and *i’* at locus *j*. Thus, the dummy variable for each haplotype were coded as: 0 = no copies of the haplotype, 1 = one copy of the haplotype (paternal or maternal), and 2 = two copies of the haplotype (paternal and maternal).

### Genome-wide association analysis

The WBSF was pre-adjusted for fixed effect of CG and age at slaughter as covariate (linear effect). Fixed effects were estimated using a single-trait animal model implemented in AIREMLF90 [26]. The univariate linear mixed model analysis was performed for pre-adjusted WBSF using single-SNPs and each size of haplotype (three, five, seven, nine or eleven SNPs) individually in GEMMA [27] using the model: *y* = 1*μ* + *Xβ* + *Zu* + *e*; *u*~*MVN*_*n*_(0, *GV*_*g*_); *e*~*MVN*_*n*_(0, *IV*_*e*_); where: *y* is an *n*-vector of pre-adjusted WBSF; *μ* is the overall mean; *X* is the incidence matrix for single SNP or haplotype; *β* is the single SNP allele or haplotype effects; *Z* is an *n* x *n* identity matrix; *u* is an *n*-vector of random residual additive genetic effects; *G* is a *n* x *n* genomic relationship matrix (GRM); *e* is a vector of residuals; *V*_*g*_ is the residual additive genetic variance component; *V*_*e*_ is the residual variance component; and *I* is an *n* x *n* identity matrix. *MVN*_*n*_ denotes the *n*-dimensional multivariate normal distribution. The GRM was estimated using the standardized genotypes for all 413,355 SNP markers retained for analysis following filtering, using GEMMA. The same GRM was used for the single-SNPs and haplotype-based association analyses.

### Estimating additive genetic variance

The additive genetic variance (V_A_) explained by SNP markers was estimated as: ${\sigma}_i^2=2{p}_i\left(1-{p}_i\right){a}_i^2$, where: ${\sigma}_i^2$ is the additive genetic variance for the *i*^th^ SNP; *p*_*i*_ is the allele frequency of one of the alleles at the *i*^th^ SNP; and *a*_*i*_ is the additive effect of the *i*^th^ SNP. An equivalent equation was used to estimate V_A_ explained by the haplotyped loci [28], as follows: ${\sigma}_j^2=\sum \limits_{i=1}^{k_j-1}\sum \limits_{l=2}^{k_j}{\left({a}_{ij}-{a}_{lj}\right)}^2{p}_{ij}{p}_{lj}$, for *l* > *i*, where: ${\sigma}_j^2$is the additive genetic variance for the *j*^th^ haplotype, *a*_*ij*_ is the additive effect of the *i*^th^ allele at the *j*^th^ haplotype, *a*_*lj*_ is the additive effect of the *l*^th^ allele at the *j*^th^ haplotype, *p*_*ij*_ is the allele frequency for the *i*^th^ allele at the *j*^th^ haplotype, *p*_*lj*_ is the allele frequency for the *l*^th^ allele at the *j*^th^ haplotype, and *k*_*j*_ is the number of existing alleles at the *j*^th^ haplotype. The additive effects and frequencies of the SNP and haplotype alleles were calculated using GEMMA software as described in section “Genome-wide association analysis”.

For the identification of the SNPs and haplotyped loci that explained the greatest amounts of V_A_ for WBSF, the estimated V_A_ were assumed to follow a gamma distribution with parameters shape (α) and rate (β) [29]. The parameters (α and β) were predicted using the V_A_ explained by the SNPs and haplotyped loci which allowed establishing the value of gamma distribution quantile corrected for multiple testing by Bonferroni method (α ≤ 0.05). For the SNPs, the α and β were predicted using an approximation of the Newton-Raphson method [30] as: $\widehat{\alpha}\approx \frac{3-s\sqrt{{\left(3-s\right)}^2+24s}}{12s}$, where: $s=\ln \left(\frac{1}{N}\sum \limits_{i=1}^N{\sigma}_i^2\right)-\frac{1}{N}\sum \limits_{i=1}^N\mathit{\ln}\left({\sigma}_i^2\right)$; $\widehat{\beta}=\frac{\widehat{a}}{\mu_{\sigma_i^2}}$, where: ${\mu}_{\sigma_i^2}$ is the mean of estimated V_A_ explained by the SNPs. For the haplotyped loci, the estimated V_A_ were dependent on the number of alleles at each haplotyped locus. Therefore, a cubic regression model was used to predict α as a function of the number of alleles. For the $\widehat{\beta}$ parameter, the Brody non-linear regression model [31] was applied, as follows: $\widehat{\beta}=A\left(1-{Be}^{- kt}\right)+\varepsilon$, where: $\widehat{\beta}$ is a predicted β parameter; *A* is the asymptotic limit for the β parameter; *B* is the integration constant; *k* is the curve parameter representing the ratio of maximum growth rate to asymptotic limit of $\widehat{\beta}$; *t* is the number of alleles at the haplotyped locus; and *ε* is the residual. From these equations, it was possible to estimate the gamma distribution in order to establish the thresholds that allowed identifying haplotyped loci (regarding the number of alleles) and SNP markers that explained the greatest amounts of V_A_ for WBSF.

### Linkage disequilibrium analysis

**Fig. 1:**
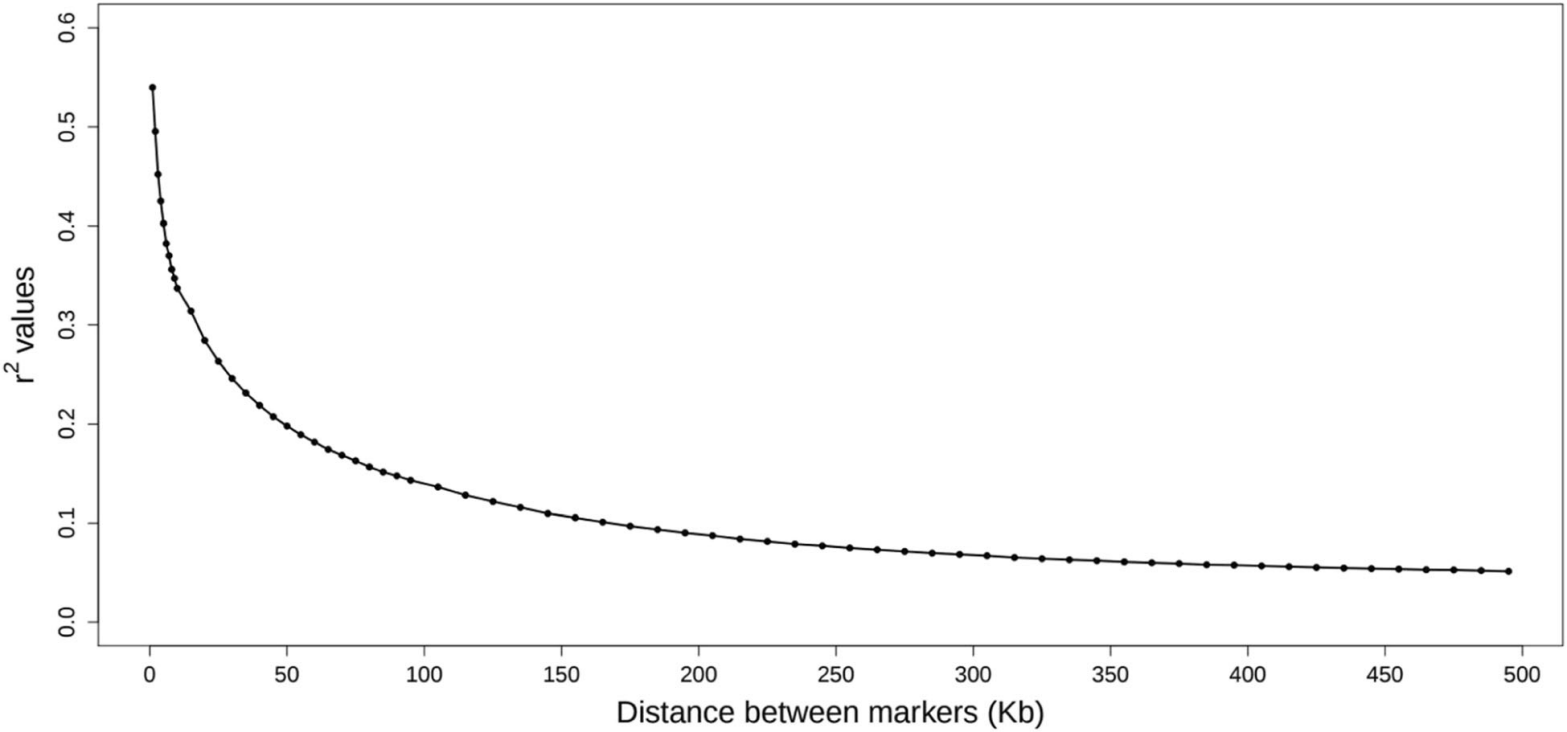
Linkage disequilibrium (r^2^) values according to distance between pairs of markers

The LD between each pair of the SNPs (413,355), measured as r^2^, was calculated using Haploview [32]. The average r^2^ values according to distance between markers are displayed in Fig. 1. The regions that explained the greatest amounts of V_A_ for WBSF were classified as strong (r^2^ > 0.6), moderate (0.2 < r^2^ < 0.6), weak (0.1 < r^2^ < 0.2), and not in LD (r^2^ < 0.1) based on the average r^2^ values between the SNPs.

### SNP and haplotype annotation and gene networks

The SNPs and the genomic regions harboring all of the haplotyped loci identified were annotated using the Variant Effect Predictor (VEP) Ensembl API [33]. The identified genes were used to predict a gene interaction network using the GeneMANIA Cytoscape plug-in [34], based on the source organism *Homo sapiens*.

## Results

### Genome-wide association analysis

The single-SNP GWAA identified eight variants influencing WBSF on BTA 3, 4, 9, 10 and 11 (Table 1). The SNP *rs134499129* (BTA3) explained the greatest amount of V_A_ (0.072 kg^**2**^) and *rs41623448* (BTA10) had the largest allele substitution effect (0.73 ± 0.09 kg). Four of the detected variants (*rs109294639*, *rs134499129*, *rs41595711* and *rs42732955*), located in *NOS1AP* and *SUCLG1* were intronic, and four variants (*rs43490295*, *rs137367597*, *rs41623448* and *rs136174419*) were intergenic. The intronic variant *rs109294639* was in moderate LD with *rs134499129* and *rs41595711* (r^**2**^ = 0.26 and 0.24, respectively), whereas *rs134499129* and *rs41595711* were in strong LD (r^**2**^ = 0.71). The intergenic variants *rs41623448* and *rs136174419* are located near *TBCD21* and *SUCLG1*, at a distance of 37 and 68 kb, respectively. The SNPs *rs136174419* (near *SUCLG1*) and *rs42732955* (*SUCLG1*, intron 1) were in strong LD (r^**2**^ = 0.79) at a distance of 74 kb. However, *rs43490295*, *rs137367597* and *rs41623448* are not in LD with any neighboring SNP markers.

**Table 1:** SNP markers that explained the greatest additive genetic variance for meat tenderness in Nelore cattle

SNP marker	BTA	Position (bp)	Allele frequencies (effects^a^)		Var (kg^2^)	Gene
*rs109294639*	3	7,384,182	*T* = 0.094 (− 0.40 ± 0.08)	C = 0.906 (0.40 ± 0.08)	0.027	*NOS1AP* (intron1)
*rs134499129*	3	7,390,035	A = 0.163 (− 0.51 ± 0.06)	G = 0.837 (0.51 ± 0.06)	0.072	*NOS1AP* (intron1)
*rs41595711*	3	7,391,544	*T* = 0.185 (− 0.30 ± 0.06)	C = 0.815 (0.30 ± 0.06)	0.027	*NOS1AP* (intron1)
*rs43490295*	4	1,008,553	G = 0.120 (− 0.37 ± 0.07)	A = 0.880 (0.37 ± 0.07)	0.028	–
*rs137367597*	9	1,116,610	C = 0.135 (−0.34 ± 0.07)	A = 0.865 (0.34 ± 0.07)	0.028	–
*rs41623448*	10	20,486,971	C = 0.064 (−0.73 ± 0.09)	*T* = 0.936 (0.73 ± 0.09)	0.063	near *TBCD21*
*rs136174419*	11	50,332,078	A = 0.112 (−0.37 ± 0.07)	G = 0.888 (0.37 ± 0.07)	0.028	near *SUCLG1*
*rs42732955*	11	50,406,682	A = 0.124 (−0.39 ± 0.07)	G = 0.876 (0.39 ± 0.07)	0.032	*SUCLG1* (intron1)

*SNP* single nucleotide polymorphism, *BTA Bos taurus* autosome, *Var* SNP marker additive genetic variance

^a^Allele substitution effects from GEMMA software (kg)

Using the haplotype-based analysis we identified haplotyped loci that explained the greatest amounts of V_A_ for WBSF (Table 2). Thirty-three putative QTL regions for WBSF were detected, with 19 being identified by at least two different haplotype-based analyses and five QTLs were identified in all of the haplotype-based analyses. The SW3, SW5, SW7, SW9 and SW11 analyses identified 57, 61, 42, 39, and 21% of all 33 putative QTL regions, respectively, whereas, the analyses using SW3 and SW5 jointly detected 88% of all 33 QTL regions. Increasing the number of SNPs included in the haplotyped loci did not always lead to an increase in the amount of V_A_ that was explained by the haplotypes. Some QTLs appeared to only be captured using larger haplotypes while other QTLs could only be detected using smaller haplotypes.

**Table 2:** QTL regions for meat tenderness detected using haplotype-based analysis on variable-sized sliding windows

BTA	Region	Position (bp)	Dist	LD	Additive genetic variance (kg^2^)					
					SNP	SW3	SW5	SW7	SW9	SW11
1	*rs43761056 – rs137207255*	6,317,864 – 6,324,488	6625	M	–	0.032	0.036	–	–	–
1	*rs42493480 – rs42493494*	125,981,534 – 126,011,455	29,922	M	–	–	0.031	0.034	0.038	–
3	*rs109872503 – rs110244139*	7,371,235 – 7,460,489	89,255	M	0.072	0.098	0.071	0.085	0.086	0.089
3	*rs132795858 – rs134534136*	29,474,206 – 29,610,884	136,679	S	–	0.038	0.049	0.051	0.051	0.050
3	*rs132763845 – rs109427593*	90,594,180 – 90,664,268	70,089	M	–	–	–	0.036	0.045	–
3	*rs134111725 – rs133976586*	93,949,186 – 94,067,553	118,368	S	–	–	–	–	–	0.040
4	*rs43490295*	1,008,553	–	N	0.028	–	–	–	–	–
4	*rs137252969 – rs43385178*	24,143,662 – 24,225,606	81,945	W	–	–	0.037	–	–	–
4	*rs110513194 – rs135604613*	27,834,564 – 27,894,006	59,443	S	–	–	–	–	0.042	–
5	*rs136407209 – rs110430223*	7,525,121 – 7,583,435	58,315	S	–	–	0.030	–	–	–
5	*rs41587994 – rs109349300*	18,434,417 – 18,465,179	30,763	S	–	0.036	0.034	–	–	–
8	*rs133253155 – rs135484797*	92,216,117 – 92,289,515	73,399	S	–	0.033	0.034	0.033	–	–
9	*rs137367597*	1,116,610	–	N	0.028	–	–	–	–	–
9	*rs110938040 – rs135589084*	12,339,368 – 12,406,450	67,083	M	–	–	–	–	0.048	0.053
9	*rs136867942 – rs135252570*	21,441,424 – 21,497,606	56,183	M	–	–	–	–	0.037	–
9	*rs133390891 – rs43600182*	61,168,282 – 61,197,874	29,593	S	–	–	0.037	–	–	–
9	*rs110730224 – rs109847831*	66,977,872 – 67,045,614	67,743	M	–	0.040	0.039	0.040	0.040	–
9	*rs135160781 – rs110936646*	100,716,451 – 100,721,452	5002	S	–	0.034	–	–	–	–
10	*rs137812088 – rs133615734*	20,448,593 – 20,541,764	93,172	M	0.063	0.040	0.041	0.038	0.044	0.052
10	*rs134006987 – rs136312573*	43,692,759 – 43,726,489	33,731	S	–	0.033	0.031	–	–	–
11	*rs109122230 – rs41655045*	5,751,331 – 5,768,629	17,318	M	–	0.033	–	–	–	–
11	*rs43755797 – rs137032372*	43,129,117 – 43,135,745	6629	M	–	0.032	–	–	–	–
11	*rs136174419 – rs42731923*	50,332,078 – 50,417,494	85,417	S	0.032	0.069	–	–	–	–
15	*rs132639440 – rs136091960*	72,439,829 – 72,470,564	30,736	M	–	–	0.036	0.040	0.051	–
17	*rs110304377 – rs42926409*	70,788,438 – 70,935,589	147,152	M	–	0.035	0.043	0.054	0.056	–
18	*rs137081181 – rs134146295*	14,849,540 – 14,991,573	142,034	S	–	0.039	0.041	0.043	0.043	0.045
24	*rs135709192 – rs136715705*	47,570,379 – 47,596,815	26,437	S	–	0.033	0.033	0.034	0.034	0.034
24	*rs110779214 – rs109148899*	48,585,933 – 48,632,298	46,366	S	–	0.038	0.038	–	–	–
24	*rs136382747– rs135235176*	55,868,854 – 55,975,740	106,887	M	–	–	–	0.040	–	–
25	*rs110607501 – rs108984883*	17,590,025 – 17,598,054	8030	S	–	0.033	0.034	0.034	–	–
26	*rs133176306 – rs109605337*	19,035,737 – 19,066,768	31,032	S	–	0.034	0.038	–	–	–
28	*rs134774253 – rs42146826*	26,876,269 – 26,885,074	8806	S	–	0.033	–	–	–	–
29	*rs136755211 – rs42192064*	43,980,089 – 44,042,363	62,275	S	–	–	0.032	0.032	–	–

*BTA Bos taurus* autosome, *Dist* distance, *LD* linkage disequilibrium, *SNP* single nucleotide polymorphism, *SW* Sliding window haplotype, *SW3* SW of three SNPs, *SW5* SW of five SNPs, *SW7* SW of seven SNPs, *SW9* Haplotype SW of nine SNPs, *SW11* SW of eleven SNPs, *S* strong LD (r^2^ > 0.6), *M* moderate LD (0.2 < r^2^ < 0.6), *W* weak LD (0.1 < r^2^ < 0.2), *N* not LD (r^2^ < 0.1)

Putative QTLs identified are located on BTA 1, 3, 4, 5, 8, 9, 10, 11, 15, 17, 18, 24, 25, 26 and 29. SNPs within most of the QTL regions found in this study were in strong or medium pair-wise LD and the length of the QTL regions varied from 5 to 147 kb with an avarage of 63 kb, as shown in Table 2. The QTL with the largest effect was identified in all of the haplotype window size analyses, however, the window for the SW3 analysis explained the greatest amount of V_A_ (0.098 kg^**2**^). This QTL region harbors *NOS1AP* (BTA3) and includes three SNPs (*rs109294639*, *rs134499129* and *rs41595711*) identified in the single-SNP GWAA.

The SNPs *rs43490295* and *rs137367597* on BTA4 and BTA9 (Table 2), respectively, were only detected by the single-SNP GWAA. On the other hand, the haplotype-based association analyses captured many QTL regions that were not identified by the single-SNP GWAA. Full results for single-SNP and haplotype-based association analyses for each number of alleles at haplotyped loci are shown in Additional files 1, 2, 3, and 4.

### Gene network analysis

**Fig. 2:**
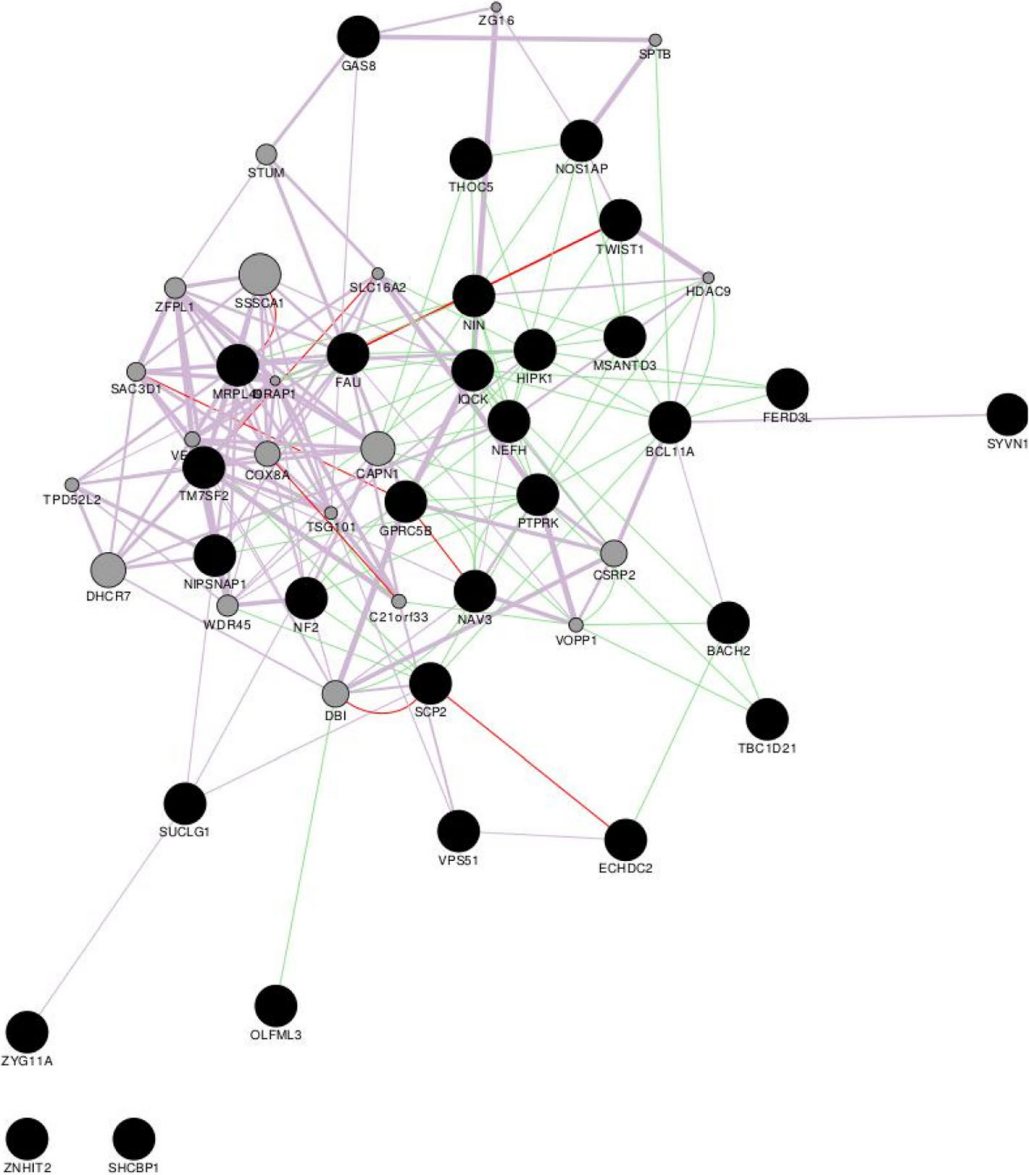
Gene interaction network for genes in QTL regions for meat tenderness (WBSF). Genes presented as black circles were located in the QTL regions and genes that interact with those as grey circles. Edges in purple, green and red represent co-expression relationships, genetic interactions and co-localizations, respectively

Figure 2 illustrates the interactions among the genes located in genomic regions detected as being influencing WBSF in this study. The constructed gene interaction network revealed that 66.7% of the constituant genes are known to be co-expressed in humans. Moreover, 26.7% of the genes co-localize indicating that they are expressed in the same tissue or that their proteins are both identified in the same cellular locations. In addition, 70% of the genes interact, suggesting that they are functionally associated. The gene interaction network also revealed 20 genes, presented as grey circles, that interact with genes in the genomic regions that cause variation in WBSF. Among these, *CAPN1* is related to genes that were found to be influencing WBSF in this study. *CAPN1* interacts with *NEFH*, *NIN*, *PTPRK* and *THOC5* and is co-expressed with *MRPL49*, *SUCLG1*, *TM7SF2* and *NF2*.

## Discussion

We performed a single-SNP GWAA and investigated a strategy for haplotype-based analysis using variable-sized sliding windows to detect genomic regions that influence WBSF. The desirable alleles (negative effects) for all eight SNPs identified influencing WBSF using single-SNP GWAA were in low frequency (Table 1), indicating that selection for these desirable alleles could improve tenderness in Nelore cattle. We found haplotypes affecting WBSF that were not detected in the single-SNP analysis (Table 2). This is consistent with previous studies that have shown that haplotype-based analysis provides a greater power for QTL detection than does single SNP analysis [15, 16, 35, 36]. The most likely reason for this finding is that QTL are more likely to be in strong LD with a multi-marker haplotype than with a single biallelic SNP, thus haplotype-based association methods have the opportunity to capture greater numbers of associations [10, 37, 38].

### Genome-wide association analysis

The single-SNP analysis detected two SNPs (*rs43490295* and *rs137367597*) that were not identified in the haplotype-based analyses. These SNPs explained the smallest amounts of QTL variance among the detected SNPs (0.028 kg^2^) and they were not in LD with neighboring SNPs. Using simulated data, [21] showed that haplotypes did not provide an advantage for detecting QTL with small effect sizes. In addition, haplotype-based analysis may also not have captured these signals because haplotype-based tests tend to be more powerful when moderate to high levels of LD exist in a chromosome region [39].

The variable-sized sliding window haplotype analysis strategy was used to ensure that every region of the genome was included in the analysis and that causal loci could be spanned by haplotyped regions. However, the number of contiguous SNPs to include in a haplotype window is a variable that requires optimization [9, 10]. The optimal haplotype size will vary according to SNP density, the patterning of LD throughout the genome and the genetic architecture of trait variation [40, 41]. We investigated five window sizes for haplotypes, containing 3, 5, 7, 9 or 11 SNP markers (SW3, SW5, SW7, SW9 and SW11, respectively). The choice of a maximum window length of 11 SNP markers was made based upon computational efficiency. The SW5 analysis detected the largest number of haplotypes loci followed by the SW3 analysis. This result may be because longer haplotype windows are more likely to introduce analytical problems such as high rates of recombination among distal SNPs resulting in excessive numbers of haplotypes, creating noise and computer memory problems [39, 42], and reducing the benefits of improved LD between haplotypes and causal variants [43].

Grapes et al.[35, 44] also found that the power to detect QTL improved as haplotype length increased to 6 SNP markers and decreased thereafter using a simulated bovine data set. However, [45] has shown that haplotypic diversity was best captured by a 20-marker sliding window in U.S. Angus cattle. The extent of LD in Nelore is much less than in Angus [46], suggesting that smaller window sizes will optimally capture haplotypic diversity in indicine cattle breeds. This assumption is supported by the length of all haplotype windows found in this study that ranged from 5 kb to 147 kb, and the SNPs within most of the QTL regions were in strong or medium pair-wise LD (Table 2). As shown in Fig. 1, the useful LD in our population extends for less than 100 kb (r^2^ < 0.15), which is consistent with the findings of [47]. This suggests that haplotypes based on sliding windows of size no more than 100 kb will capture the LD genome-wide. On the other hand, some putative QTLs were detected only by larger haplotypes. One possible explanation is that such QTL have small to very small effects on WBSF, therefore many informative SNPs grouped would have larger aggregated effects [22]. Other hypothesis is that only the allele frequencies of the larger haplotypes were similar to the QTL allele frequencies in these regions, which allowed their detection [21, 38]. Thus, since the genetic architecture and population history differ across genes and traits, it is not reasonable to expect that one single method would be superior at detection of all QTL [21].

### Genes influencing WBSF

The SNP and haplotype-based association analyses identified 37 candidate genes influencing WBSF in Nelore cattle (Table 3). Among these, five genes (*GAS8*, *OLFML3*, *TWIST1*, *GPRC5B*, and *HIPK1*) are involved in myogenesis, which is responsible for generating muscle tissue during embryonic development, regeneration of the mature skeletal musculature and maintenance of tissue homeostasis [48–52].

**Table 3:** Genes located in QTL regions for meat tenderness in Nelore cattle

BTA	Position (bp)	Genes	Gene Name	Gene type
1	6,317,864 – 6,324,488	–	*–*	–
1	125,981,534 – 126,011,455	–	*–*	–
3	7,371,235 – 7,460,489	ENSBTAG00000010158	*NOS1AP*	Protein coding
3	29,474,206 – 29,610,884	ENSBTAG00000024319	*LOC107132293*	Pseudogene
		ENSBTAG00000011322	*HIPK1*	Protein coding
		ENSBTAG00000011327	*OLFML3*	Protein coding
3	90,594,180 – 90,664,268	–	*–*	–
3	93,949,186 – 94,067,553	ENSBTAG00000002910	*ECHDC2*	Protein coding
		ENSBTAG00000003746	*SCP2*	Protein coding
		ENSBTAG00000026032	*FERD3L*	Protein coding
4	1,008,553	–	*–*	–
4	24,143,662 – 24,225,606	ENSBTAG00000010168	*–*	–
4	27,834,564 – 27,894,006	ENSBTAG00000039955	*ZYG11A*	Protein coding
		ENSBTAG00000046922	*TWIST1*	Protein coding
5	7,525,121 – 7,583,435	ENSBTAG00000009852	*NAV3*	Protein coding
5	18,434,417 – 18,465,179	–	*–*	–
8	92,216,117 – 92,289,515	–	*–*	–
9	1,116,610	–	*–*	–
9	12,339,368 – 12,406,450	-	-	-
9	21,441,424 – 21,497,606	–	*–*	–
9	61,168,282 – 61,197,874	ENSBTAG00000020713	*BACH2*	Protein coding
9	66,977,872 – 67,045,614	ENSBTAG00000020829	*PTPRK*	Protein coding
9	100,716,451 – 100,721,452	–	*–*	–
10	20,448,593 – 20,541,764	ENSBTAG00000007800	*TBC1D21*	Protein coding
10	43,692,759 – 43,726,489	ENSBTAG00000020281	*NIN*	Protein coding
11	5,751,331 – 5,768,629	ENSBTAG00000042179	*U6*	SnRNA
		ENSBTAG00000046971	*LOC107131213*	Protein coding
11	43,129,117 – 43,135,745	ENSBTAG00000016534	*BCL11A*	Protein coding
11	50,332,078 – 50,417,494	ENSBTAG00000006075	*SUCLG1*	Protein coding
		ENSBTAG00000042444	*U6*	SnRNA
15	72,439,829 – 72,470,564	ENSBTAG00000037580	*MSANTD3*	Protein coding
17	70,788,438 – 70,935,589	ENSBTAG00000013150	*THOC5*	Protein coding
		ENSBTAG00000013153	*NF2*	Protein coding
		ENSBTAG00000013152	*NIPSNAP1*	Protein coding
		ENSBTAG00000013147	*NEFH*	Protein coding
18	14,849,540 – 14,991,573	ENSBTAG00000007096	*GAS8*	Protein coding
		ENSBTAG00000025283	*LOC101904595*	Pseudogene
		ENSBTAG00000029640	*U1*	SnRNA
		ENSBTAG00000033441	*SHCBP1*	Protein coding
24	47,570,379 – 47,596,815	–	*–*	–
24	48,585,933 – 48,632,298	–	*–*	–
24	55,868,854 – 55,975,740	–	*–*	–
25	17,590,025 – 17,598,054	ENSBTAG00000019596	*GPRC5B*	Protein coding
		ENSBTAG00000044092	*IQCK*	Protein coding
26	19,035,737 – 19,066,768	–	*–*	–
28	26,876,269 – 26,885,074	–	*–*	–
29	43,980,089 – 44,042,363	ENSBTAG00000005069	*TM7SF2*	Protein coding
		ENSBTAG00000005073	*ZNHIT2*	Protein coding
		ENSBTAG00000005075	*MRPL49*	Protein coding
		ENSBTAG00000005076	*SYVN1*	Protein coding
		ENSBTAG00000015499	*VPS51*	Protein coding
		ENSBTAG00000020807	*FAU*	Protein coding

Several genes located in the QTL regions for WBSF, such as *NEFH*, *FERD3L*, *NAV3*, *BCL11A*, *ZNHIT2*, *NOS1AP* and *SHCBP1*, participate in neurogenesis processes (GO:0022008) or the structure and function of neurons. Neurogenesis is essential for skeletal muscle development and regeneration [53]. Motor neuron, a neuromuscular junction component, regulates skeletal muscle contraction [54] and also has a role in the development and differentiating of muscle fibers [55]. *NEFH*, *SHCBP1* and *NOS1AP* have previously been associated with WBSF in Nelore steers [6]. Moreover, *NOS1AP* has been associated with *longissimus* muscle area and marbling score in cattle [56]. *BCL11A* has been associated with marbling score in Canchim beef cattle [57] and a QTL region for meat tenderness harbored *ZNHIT2* in pigs [58].

The *NIN* and *NF2* genes putatively affect skeletal muscle structure and composition. *NIN* encodes a microtubule nucleation protein, which has previously been associated with skeletal muscle differentiation [59]. *NF2* participates in actin cytoskeleton organization (GO:0030036), which may explain the association with WBSF after 7 days of aging in Nelore steers found by [6]. This process results in the assembly, arrangement of constituent parts, or disassembly of cytoskeletal structures comprising actin filaments and their associated proteins (GO:0030036). Actin is a myofibril protein, one of the major components of the sarcomere, which has been reported to affect meat tenderness [60].

Genes involved in lipid and fatty acid metabolism, such as *SUCLG1*, *THOC5*, *NIPSNAP1*, *IQCK*, *TM7SF2*, *VPS51*, *PTPRK*, *ECHDC2* and *SCP2*, were also found to influence meat tenderness. Positive effects of lipid on meat tenderness are likely due to the lipid within cells in the perimysium, which separate muscle fiber bundles [61]. Furthermore, [62] found a genetic correlation between meat tenderness and fatty acid abundance using animals from the same population as used in the present study. *THOC5* was found to be differentially expressed between low and high marbling beef cattle in a *longissimus dorsi* muscle transcriptome analysis [63]. [58] reported that *TM7SF2* and *VPS51* were located within a QTL region for meat tenderness in pigs. The protein encoded by *PTPRK* has been associated with marbling in beef cattle [64]. *ECHDC2* expression is negatively correlated with non-esterified fatty acid abundance in pigs [65]. *SCP2* was down-regulated in pork with high intramuscular fat content [66] and was associated with WBSF after 7 and 14 days of aging in Nelore steers [6].

There was no evidence of a biological link or biological mechanism connecting *TBC1D21*, *MSANTD3*, *MRPL49*, *SYVN1*, *FAU*, *ZYG11A* or *BACH2* genes to meat tenderness. These genes may play a role in the regulation of transcription or translation, or may interact with important genes for meat tenderness as shown in Fig. 2. *TBC1D21* encodes a GTPase-activating protein (GO:0090630) for Rab family proteins, which are involved in spermatogenesis [67]. Its association with meat tenderness appears to be through interactions with *BACH2* and *PTPRK*. *MSANTD3* is a member of the MSANTD3 family which contains DNA binding domains for Myb proteins and the SANT domain family. [68] speculated that *MSANTD3* may be a transcription factor. In addition, *MSANTD3* interacts with *BCL11A*, *NIN*, *NOS1AP*, *SCP2* and *TWIST1*. A QTL region for meat tenderness in pigs harbors *MRPL49*, *SYVN1* and *FAU* [58]. *ZYG11A* encodes a protein that plays an important role in the regulation of ubiquitin-protein transferase activity (GO:0051438). A ZYG11A family member has been related to marbling in beef cattle [63]. *BACH2* is a transcription repressor and plays essential roles in the regulation of B cell development. B cells function in the humoral immunity component of the adaptive immune system by secreting antibodies [69]. *BACH2* has been related to intramuscular fat content in bulls [70] and was associated with WBSF in Nelore steers [6].

### Gene network analysis

The gene network analysis (Fig. 2) revealed interactions among the genes within QTL regions for WBSF and other genes that were not detected as influencing WBSF in the present study (grey circles), which are enriched for their functions in the regulation of transcription (GO:0006355), cell differentiation (GO:0030154), lipid metabolic process (GO:0006629), regulation of angiogenesis (GO:0045766), thyroid hormone transport (GO:0070327), proteolysis (GO:0006508), cellular response to insulin stimulus (GO:0032869) and cytoskeleton organization (GO:0007010). Among these, is a well-known gene influencing WBSF, *CAPN1*, which is responsible for the *postmortem* breakdown of myofibrillar proteins and seems to be the primary enzyme involved in the *postmortem* tenderization process [71]. The *CAPN1* interacts with *TM7SF2*, *ZNHIT2*, *MRPL49*, *SYVN1*, *VPS51* and *FAU* genes that are located in a QTL region for meat tenderness in this study. In addition, all of these genes are located near *CAPN1* on BTA29 and their SNPs are in strong LD with SNPs located in *CAPN1* in this population (with maximum r^2^ = 0.86).

## Conclusions

This study demonstrates that GWAA using haplotypes based on variable-sized sliding windows provides substantially more power to detect QTL than does single-SNP analysis, suggesting that this methodology should be considered for genomic predictions for WBSF and other traits. Analyses performed with smaller haplotype windows (3 and 5 SNPs) detected a higher proportion of QTLs than the analyses that used larger SNP windows. However, no single sliding window analysis identified all of the QTL that were found in the analyses using window sizes from 3 to 11 SNPs. This suggests that haplotype-based GWAA should employ several window sizes in order to detect the largest number of putative QTL. Likewise, the single-SNP analysis found two putative QTL that were not found by the haplotype-based analyses. While these may be type I errors, they may also be regulatory variants.

We identified thirty-seven candidate genes influencing meat tenderness that participate in myogenesis, neurogenesis, lipid and fatty acid metabolism and skeletal muscle structure or composition processes. These findings contribute to a better understanding of the biological mechanisms underlying meat tenderness in Nelore cattle. Further validation of these genes and polymorphisms in different populations would contribute to their use in breeding programs for Nelore cattle.

## Additional files

Additional file 1: Manhattan plot for single-SNP genome-wide association analysis. (PDF 55 kb)

Additional file 2: QQ plot for single-SNP genome-wide association analysis. (PDF 14 kb)

Additional file 3: Manhattan plots for haplotype-based association analyses for each number of alleles at haplotyped loci. (PDF 8648 kb)

Additional file 4: QQ plots for haplotype-based association analyses for each number of alleles at haplotyped loci. (PDF 2144 kb)

## Abbreviations

SNP: Single nucleotide polymorphism
GWAA: Genome-wide association analysis
LD: Linkage disequilibrium
QTL: Quantitative trait locus
CG: Contemporary group
WBSF: Warner-Bratzler shear force
SW3: Sliding window haplotypes of three SNPs
SW5: Sliding window haplotypes of five SNPs
SW7: Sliding window haplotypes of seven SNPs
SW9: Sliding window haplotypes of nine SNPs
SW11: Sliding window haplotypes of eleven SNPs
GRM: Genomic relationship matrix
VEP: Variant effect predictor
BTA: *Bos taurus* autosome
NO: Nitric-oxide
nNOS: Neuronal nitric oxide synthase
HDL: High-density lipoprotein
GO: Gene ontology
