# Supplementary figures and images for: Sliding window haplotype approaches overcome single SNP analysis limitations in identifying genes for meat tenderness in Nelore cattle
Source: BMC Genet. 2019 Jan 14;20:8. doi: 10.1186/s12863-019-0713-4 (PMC6332854; doi:10.1186/s12863-019-0713-4)

Additive Genetic Variance

0.10  
0.08  
0.06  
0.04  
0.02  
0.00

1 2 3 4 5 6 7 8 9 10 11 12 13 14 15 16 17 19 21 23 25 27 29

Chromosome

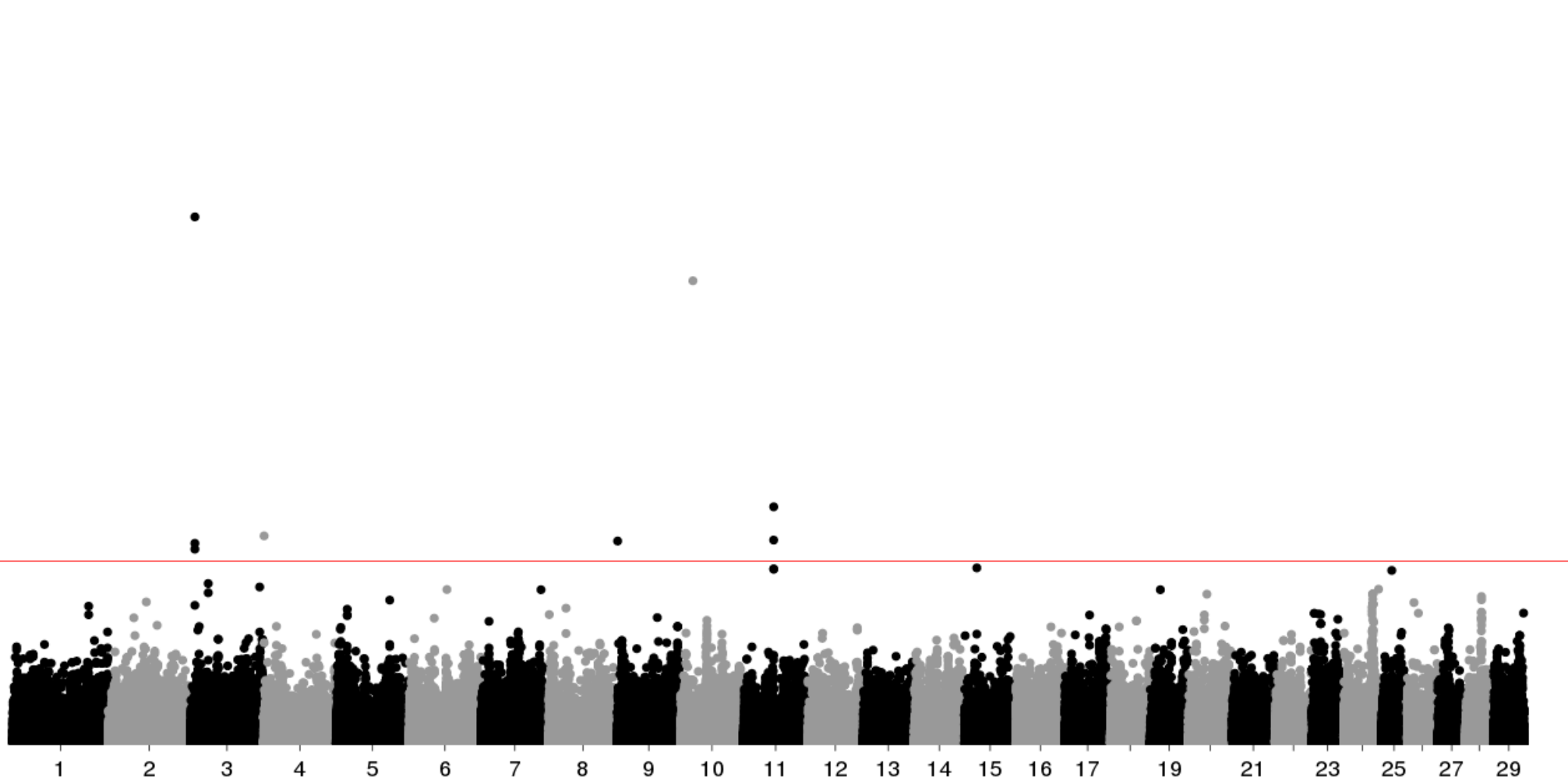

Supplement: Supplementary file 1 — Manhattan plot for single-SNP genome-wide association analysis. (PDF 55 kb) [file 12863_2019_713_MOESM1_ESM.pdf]

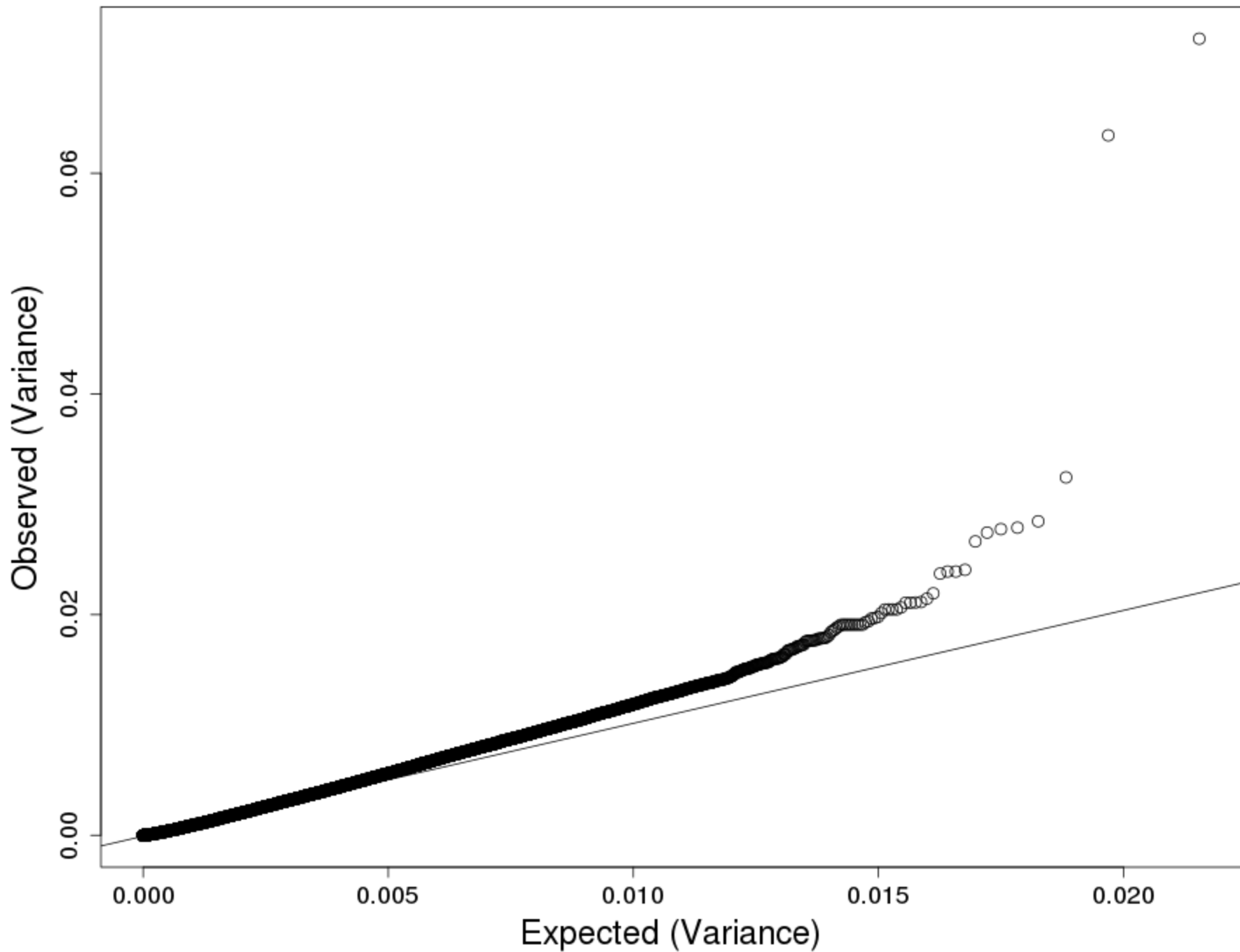

Supplement: Supplementary file 2 — QQ plot for single-SNP genome-wide association analysis. (PDF 14 kb) [file 12863_2019_713_MOESM2_ESM.pdf]
