# Supplementary material for: Sliding window haplotype approaches overcome single SNP analysis limitations in identifying genes for meat tenderness in Nelore cattle
Source: BMC Genet. 2019 Jan 14;20:8. doi: 10.1186/s12863-019-0713-4 (PMC6332854; doi:10.1186/s12863-019-0713-4)
Supplement: Supplementary file 3 — Manhattan plots for haplotype-based association analyses for each number of alleles at haplotyped loci. (PDF 8648 kb) [file 12863_2019_713_MOESM3_ESM.pdf]

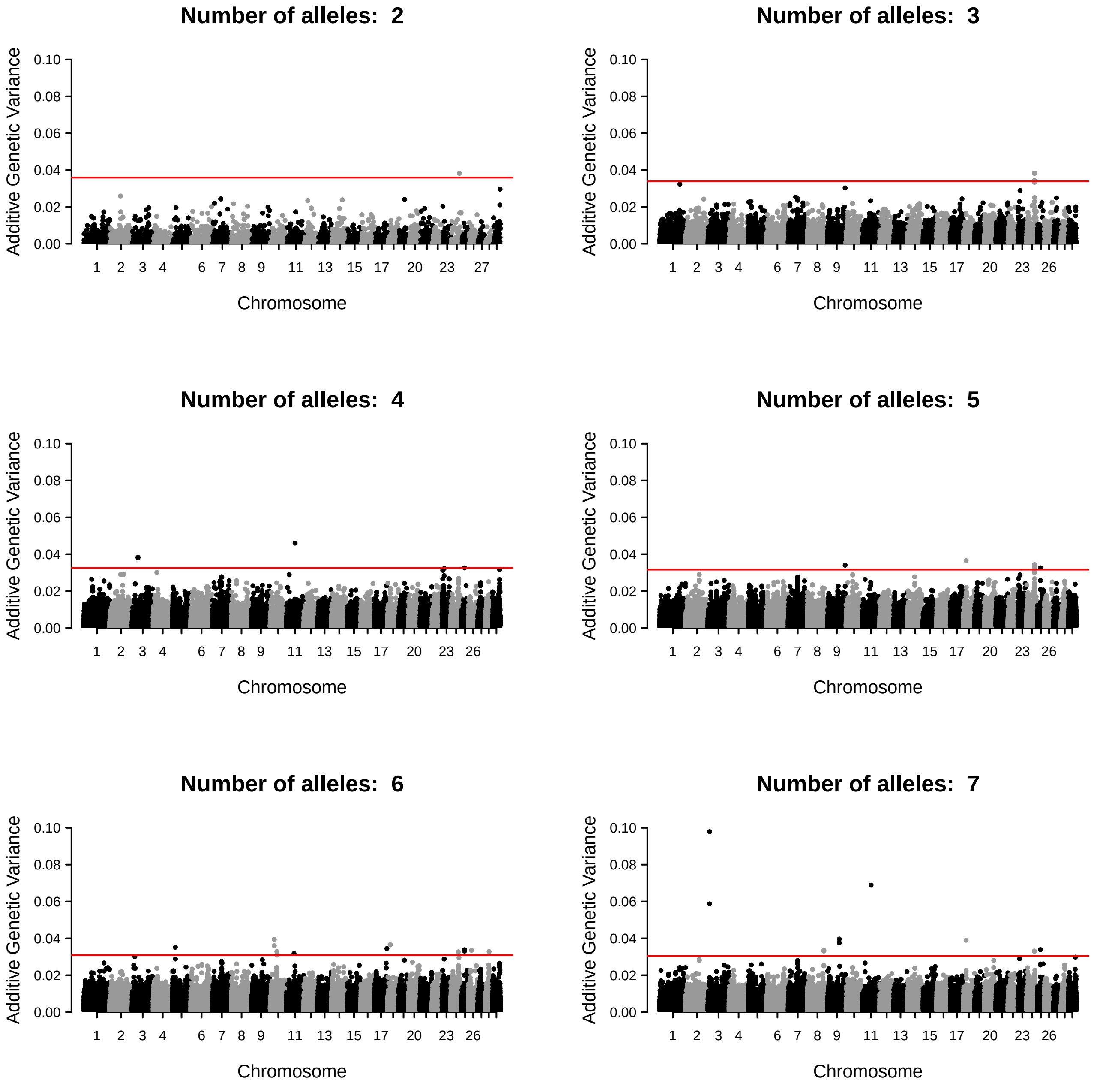
Number of alleles: 2
Number of alleles: 3
Number of alleles: 4
Number of alleles: 5
Number of alleles: 6
Number of alleles: 7
Additive Genetic Variance
0.00 0.02 0.04 0.06 0.08 0.10
Chromosome
1 2 3 4 6 7 8 9 11 13 15 17 20 23 27
1 2 3 4 6 7 8 9 11 13 15 17 20 23 26

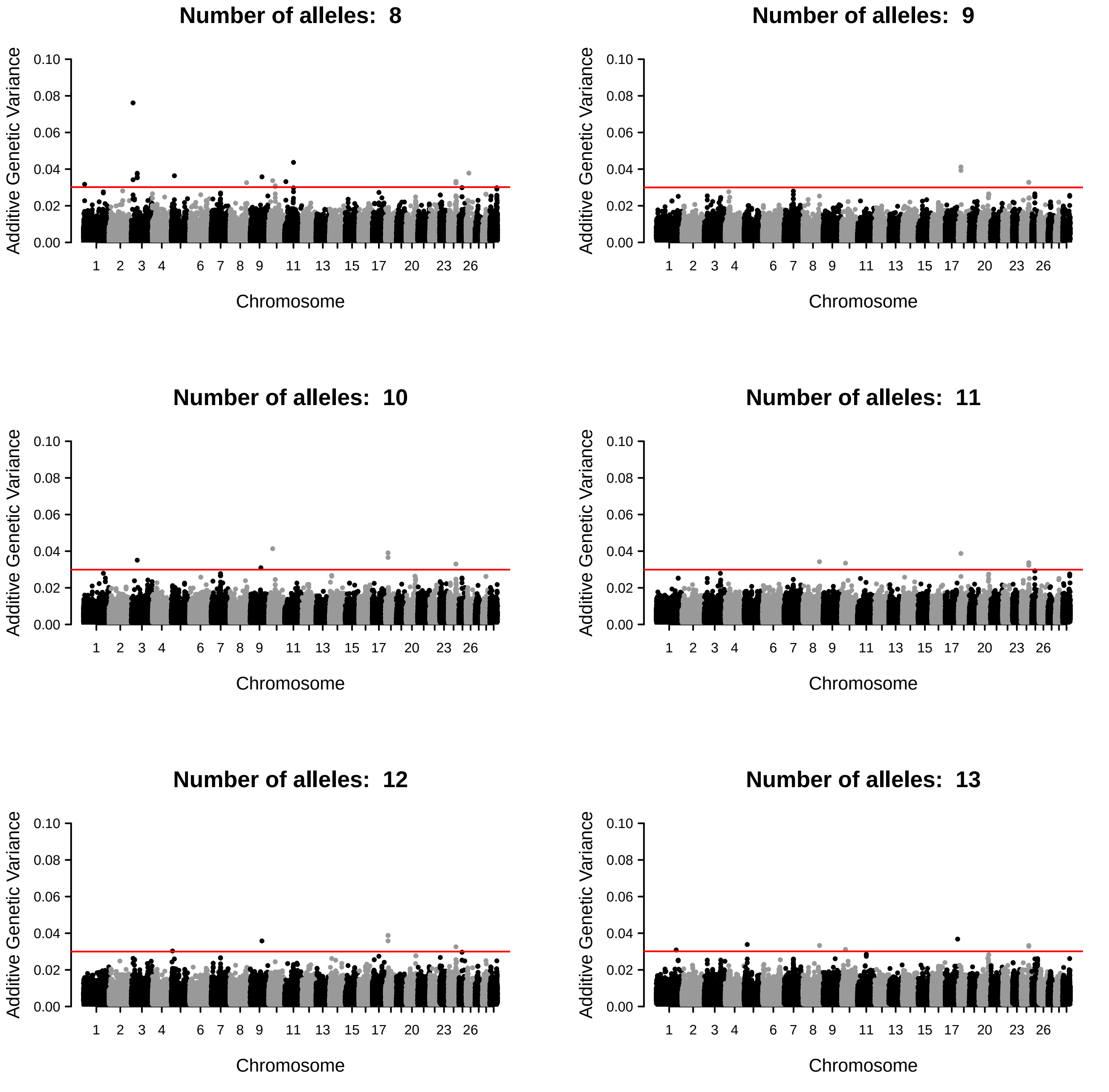

Number of alleles: 8
Number of alleles: 9
Number of alleles: 10
Number of alleles: 11
Number of alleles: 12
Number of alleles: 13
Additive Genetic Variance
0.00
0.02
0.04
0.06
0.08
0.10
Chromosome
1 2 3 4 6 7 8 9 11 13 15 17 20 23 26

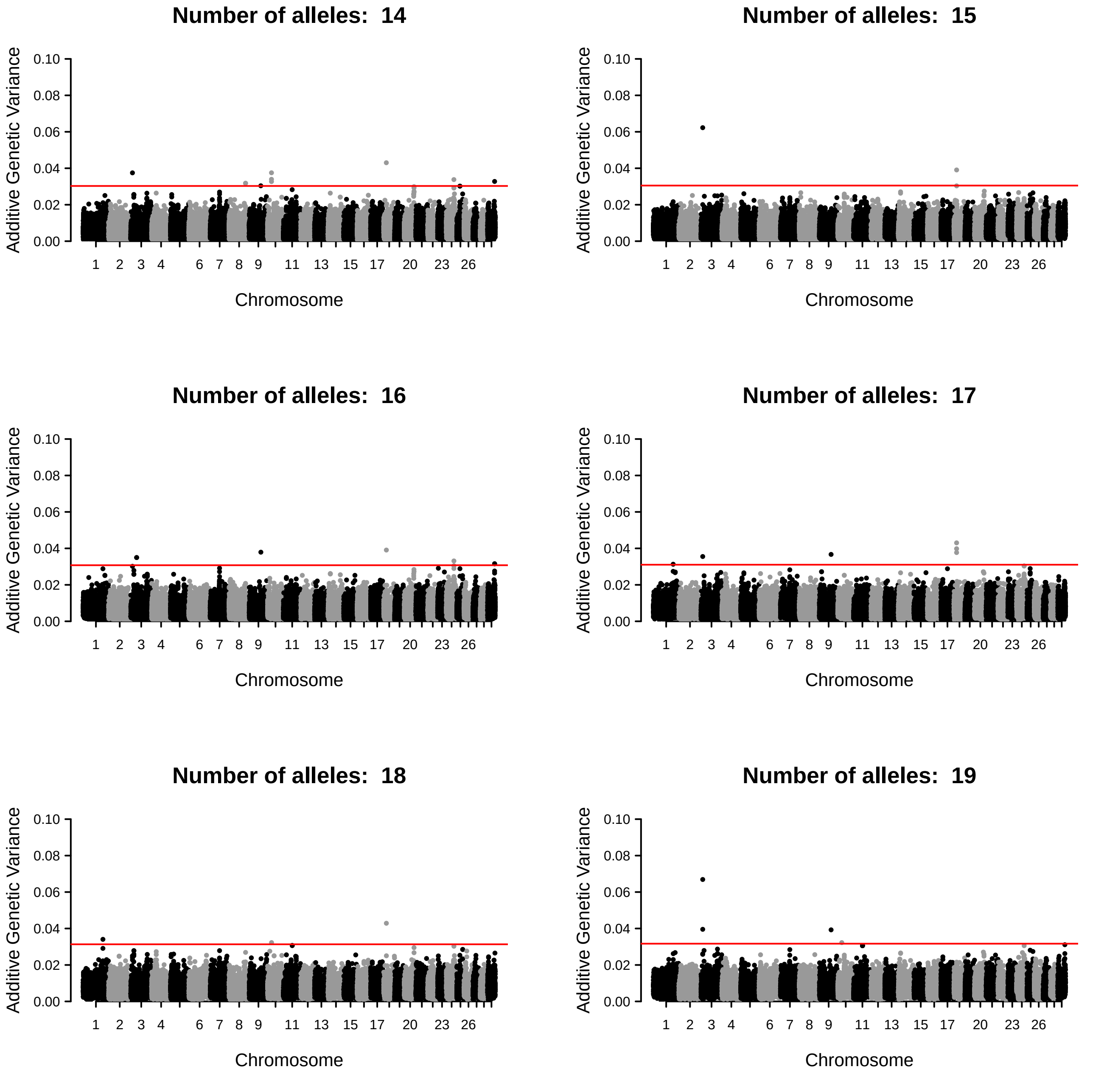
Number of alleles: 14
Number of alleles: 15
Number of alleles: 16
Number of alleles: 17
Number of alleles: 18
Number of alleles: 19
Additive Genetic Variance
0.00
0.02
0.04
0.06
0.08
0.10
Chromosome
1 2 3 4 6 7 8 9 11 13 15 17 20 23 26

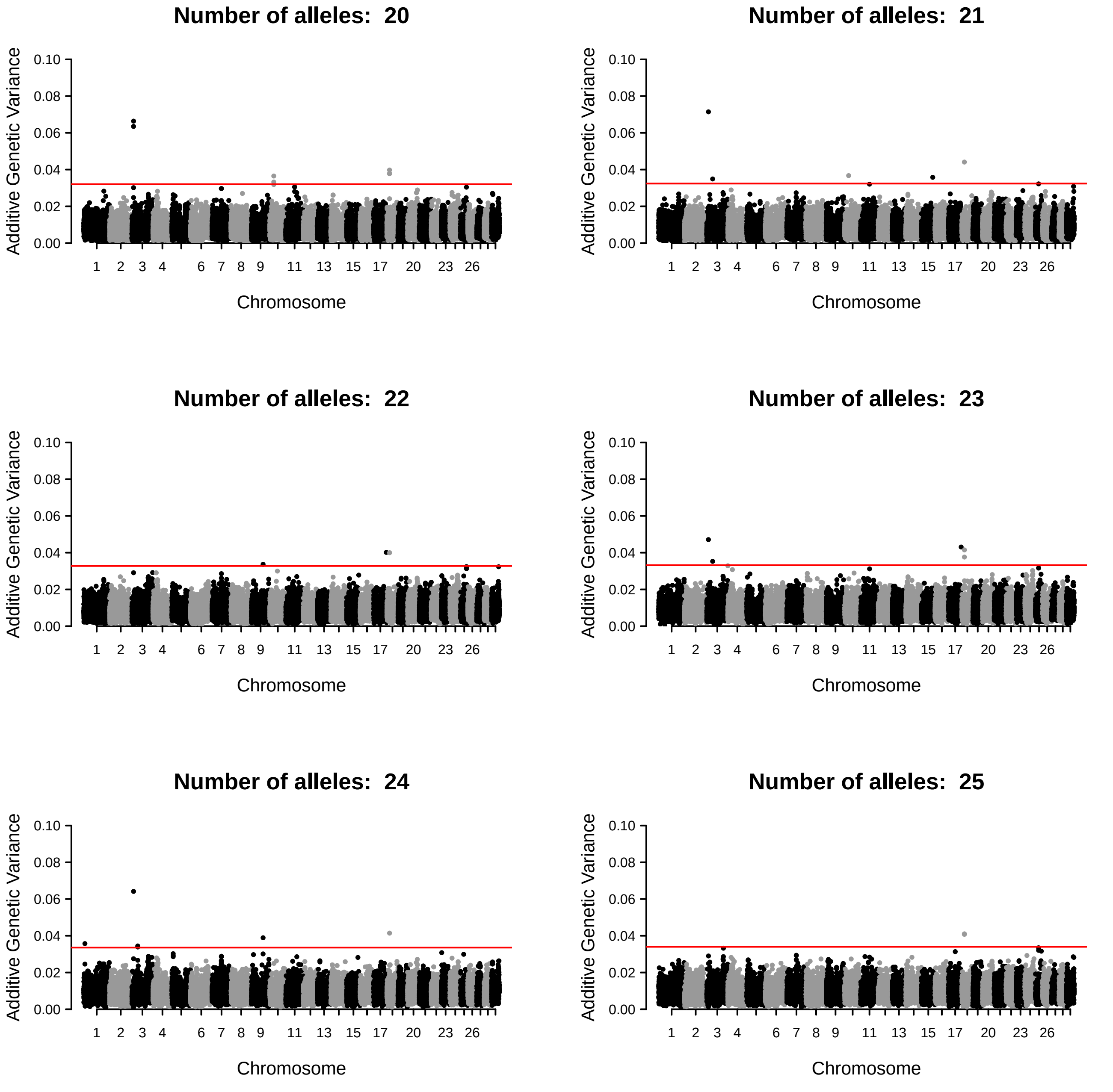
Number of alleles: 20
Number of alleles: 21
Number of alleles: 22
Number of alleles: 23
Number of alleles: 24
Number of alleles: 25
Additive Genetic Variance
0.00
0.02
0.04
0.06
0.08
0.10
Chromosome
1 2 3 4 6 7 8 9 11 13 15 17 20 23 26

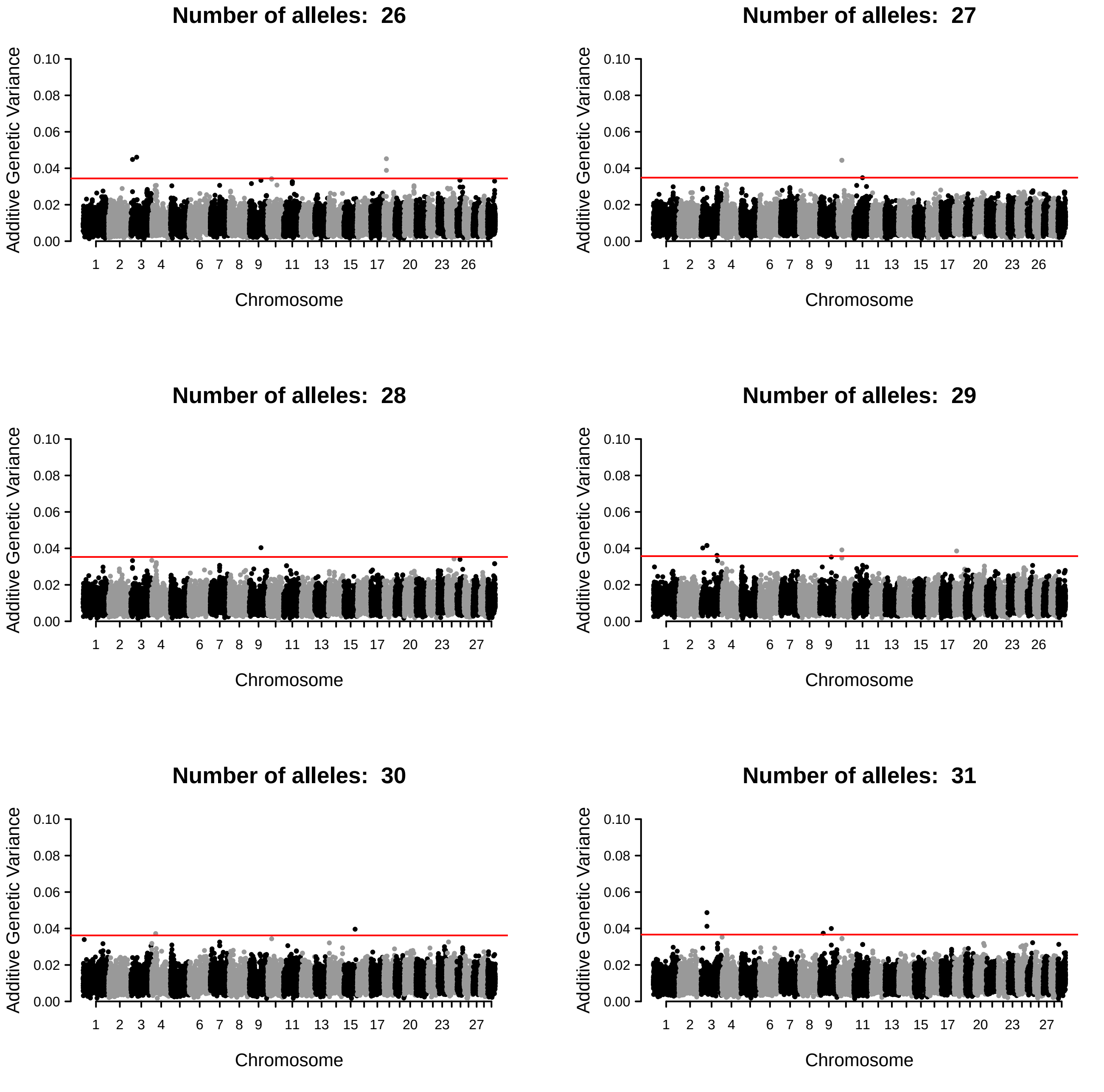

Number of alleles: 26
Number of alleles: 27
Number of alleles: 28
Number of alleles: 29
Number of alleles: 30
Number of alleles: 31
Additive Genetic Variance
0.00
0.02
0.04
0.06
0.08
0.10
Chromosome
1 2 3 4 6 7 8 9 11 13 15 17 20 23 26
1 2 3 4 6 7 8 9 11 13 15 17 20 23 27

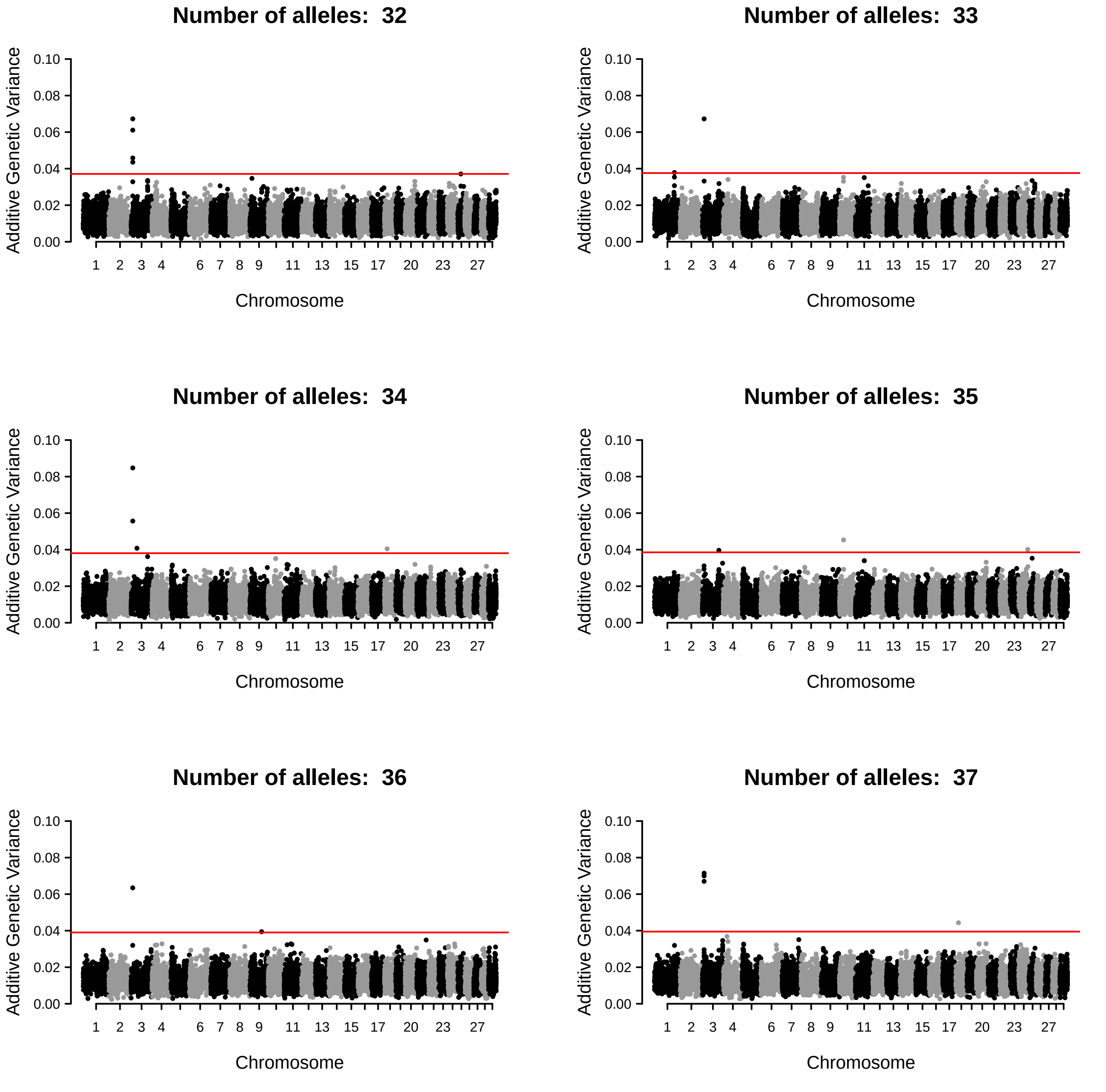
Number of alleles: 32
Number of alleles: 33
Number of alleles: 34
Number of alleles: 35
Number of alleles: 36
Number of alleles: 37
Additive Genetic Variance
0.10
0.08
0.06
0.04
0.02
0.00
1 2 3 4 6 7 8 9 11 13 15 17 20 23 27
Chromosome

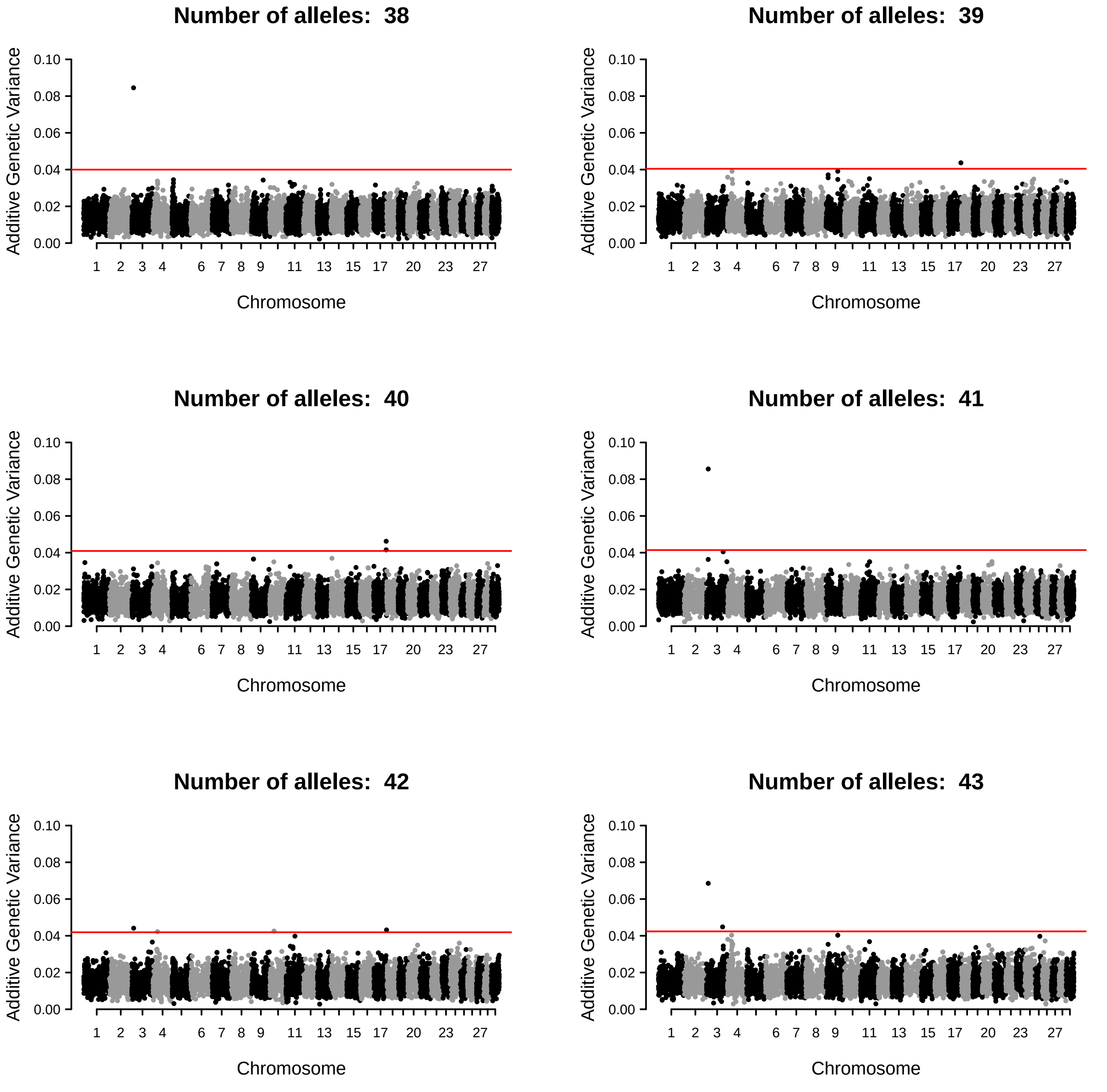

Number of alleles: 38
Number of alleles: 39
Number of alleles: 40
Number of alleles: 41
Number of alleles: 42
Number of alleles: 43
Additive Genetic Variance
0.00
0.02
0.04
0.06
0.08
0.10
Chromosome
1
2
3
4
6
7
8
9
11
13
15
17
20
23
27

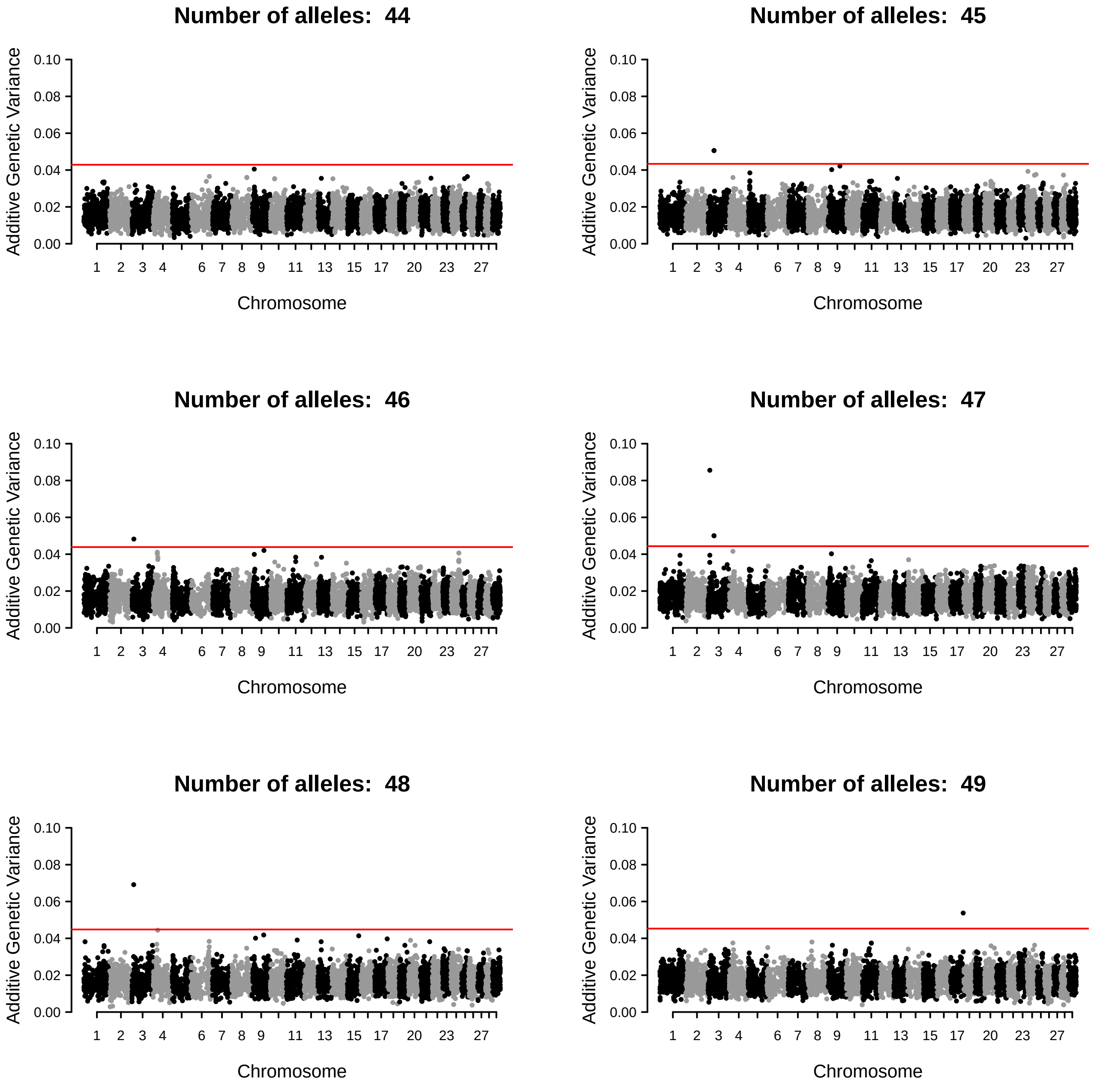
Number of alleles: 44
Additive Genetic Variance
0.10
0.08
0.06
0.04
0.02
0.00
1 2 3 4 6 7 8 9 11 13 15 17 20 23 27
Chromosome
Number of alleles: 45
Additive Genetic Variance
0.10
0.08
0.06
0.04
0.02
0.00
1 2 3 4 6 7 8 9 11 13 15 17 20 23 27
Chromosome
Number of alleles: 46
Additive Genetic Variance
0.10
0.08
0.06
0.04
0.02
0.00
1 2 3 4 6 7 8 9 11 13 15 17 20 23 27
Chromosome
Number of alleles: 47
Additive Genetic Variance
0.10
0.08
0.06
0.04
0.02
0.00
1 2 3 4 6 7 8 9 11 13 15 17 20 23 27
Chromosome
Number of alleles: 48
Additive Genetic Variance
0.10
0.08
0.06
0.04
0.02
0.00
1 2 3 4 6 7 8 9 11 13 15 17 20 23 27
Chromosome
Number of alleles: 49
Additive Genetic Variance
0.10
0.08
0.06
0.04
0.02
0.00
1 2 3 4 6 7 8 9 11 13 15 17 20 23 27
Chromosome

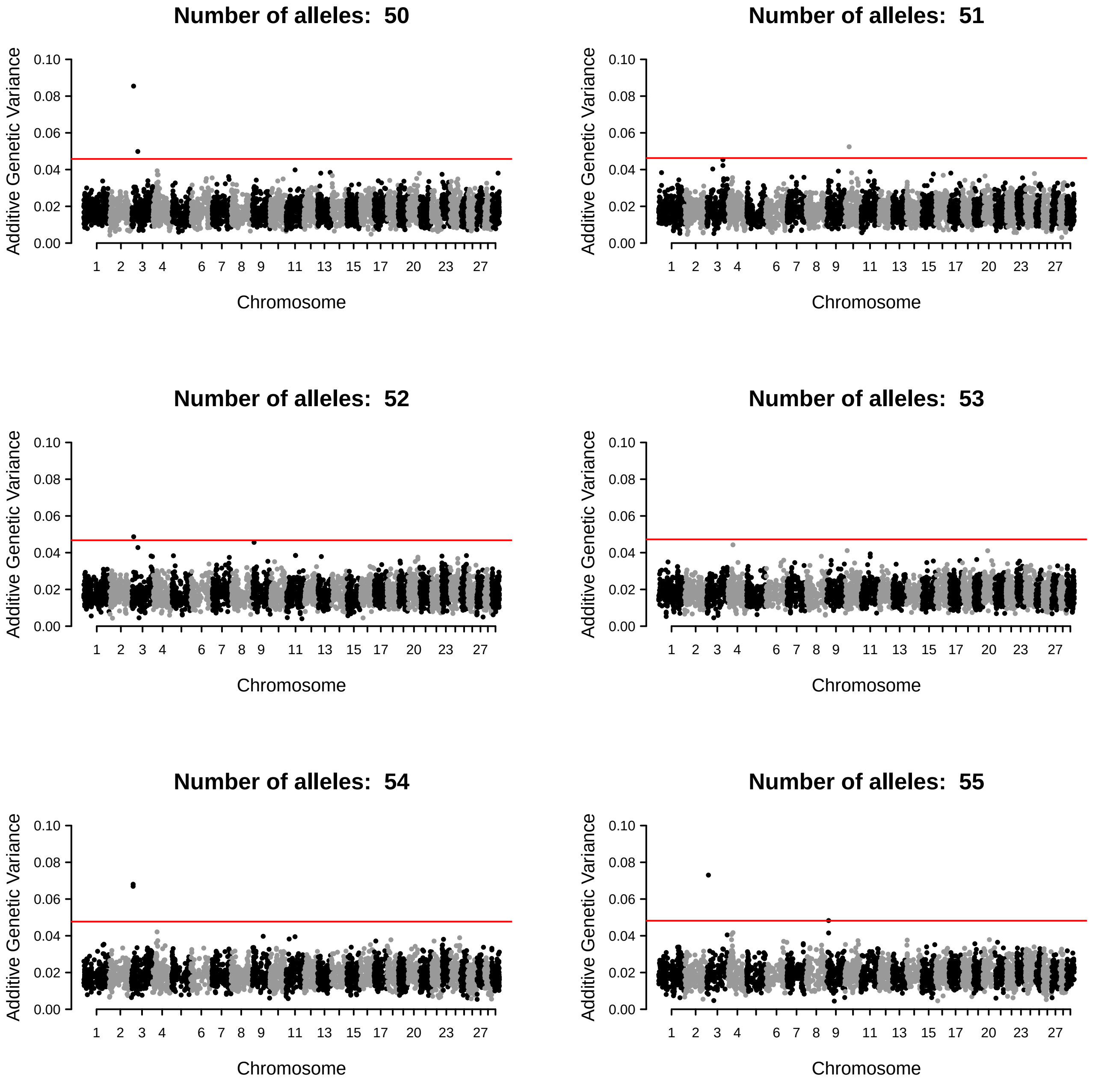
Number of alleles: 50
Number of alleles: 51
Number of alleles: 52
Number of alleles: 53
Number of alleles: 54
Number of alleles: 55
Additive Genetic Variance
0.00
0.02
0.04
0.06
0.08
0.10
1 2 3 4 6 7 8 9 11 13 15 17 20 23 27
Chromosome

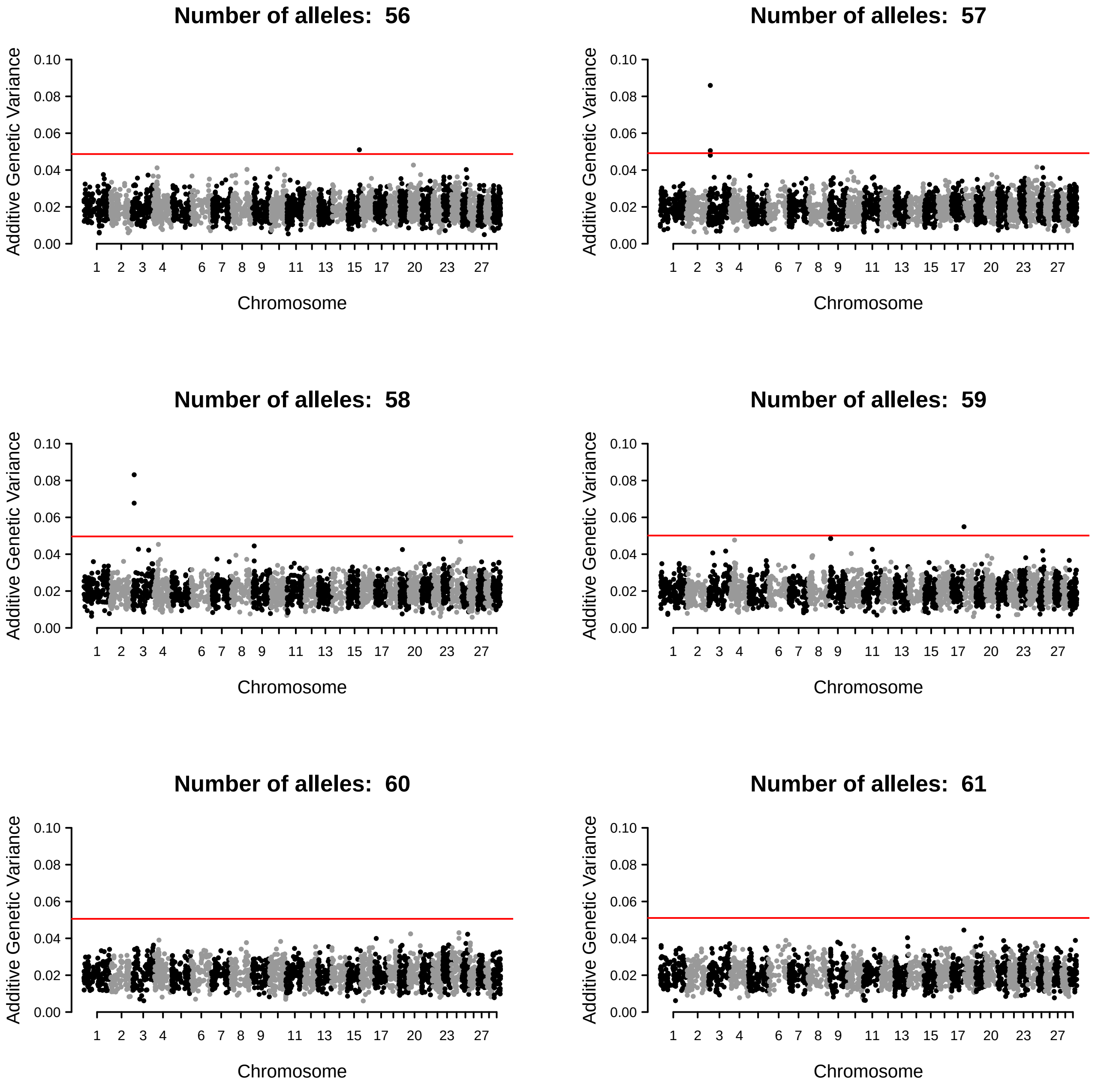
Number of alleles: 56
Number of alleles: 57
Number of alleles: 58
Number of alleles: 59
Number of alleles: 60
Number of alleles: 61
Additive Genetic Variance
0.00
0.02
0.04
0.06
0.08
0.10
Chromosome
1 2 3 4 6 7 8 9 11 13 15 17 20 23 27

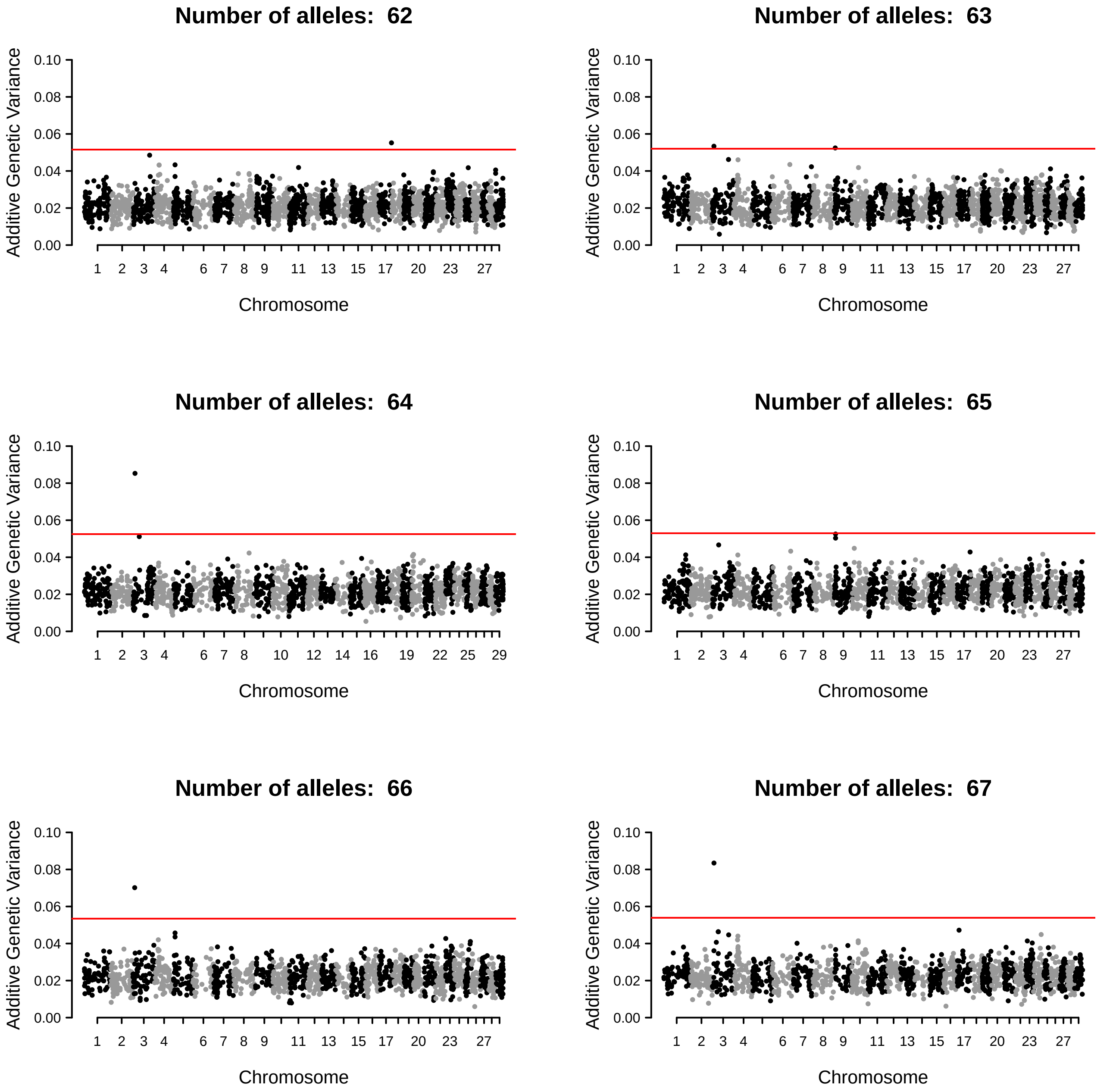
Number of alleles: 62
Number of alleles: 63
Number of alleles: 64
Number of alleles: 65
Number of alleles: 66
Number of alleles: 67
Additive Genetic Variance
0.00
0.02
0.04
0.06
0.08
0.10
Chromosome
1 2 3 4 6 7 8 9 11 13 15 17 20 23 27
1 2 3 4 6 7 8 10 12 14 16 19 22 25 29

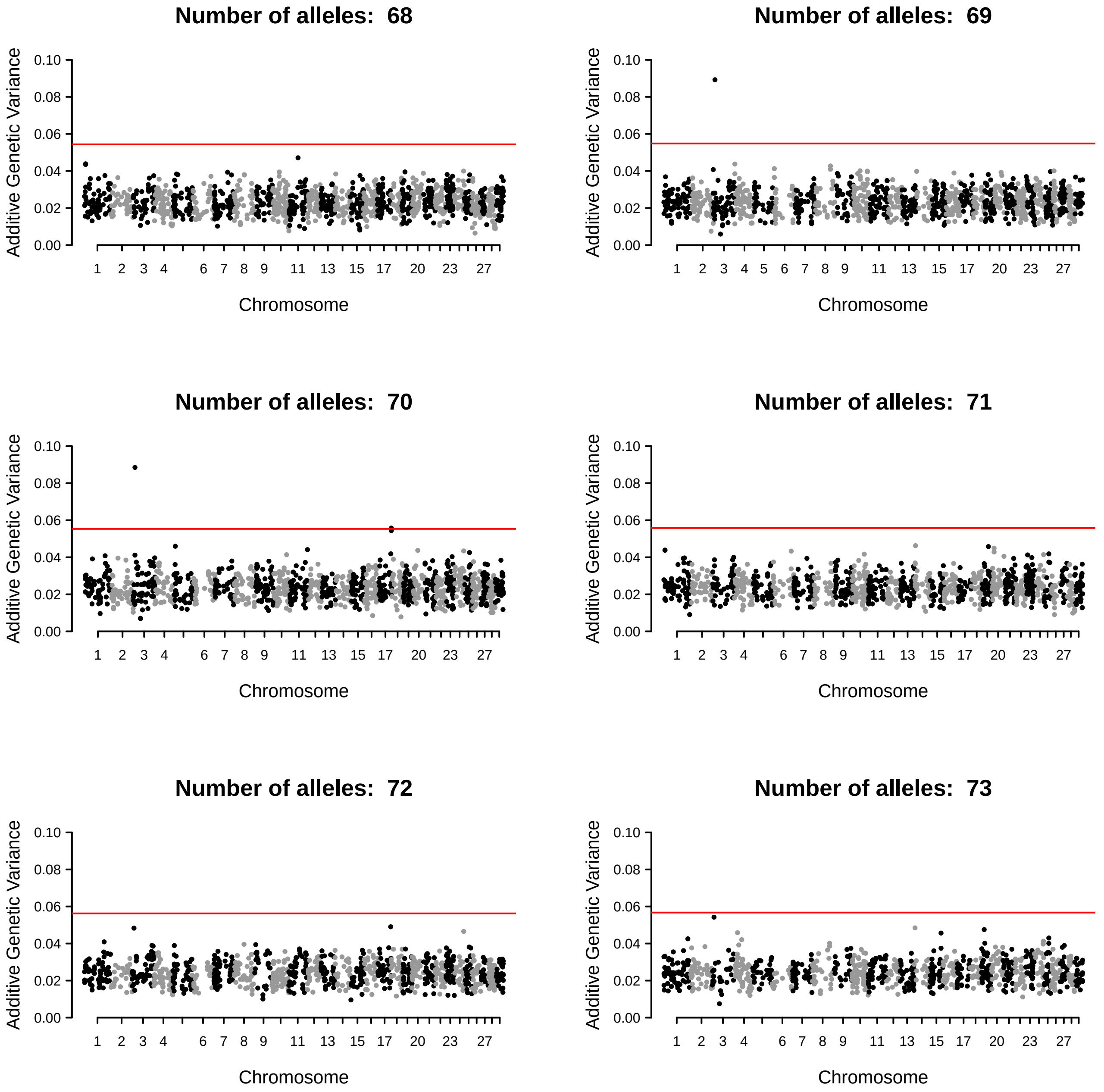

Number of alleles: 68
Number of alleles: 69
Number of alleles: 70
Number of alleles: 71
Number of alleles: 72
Number of alleles: 73
Additive Genetic Variance
Chromosome

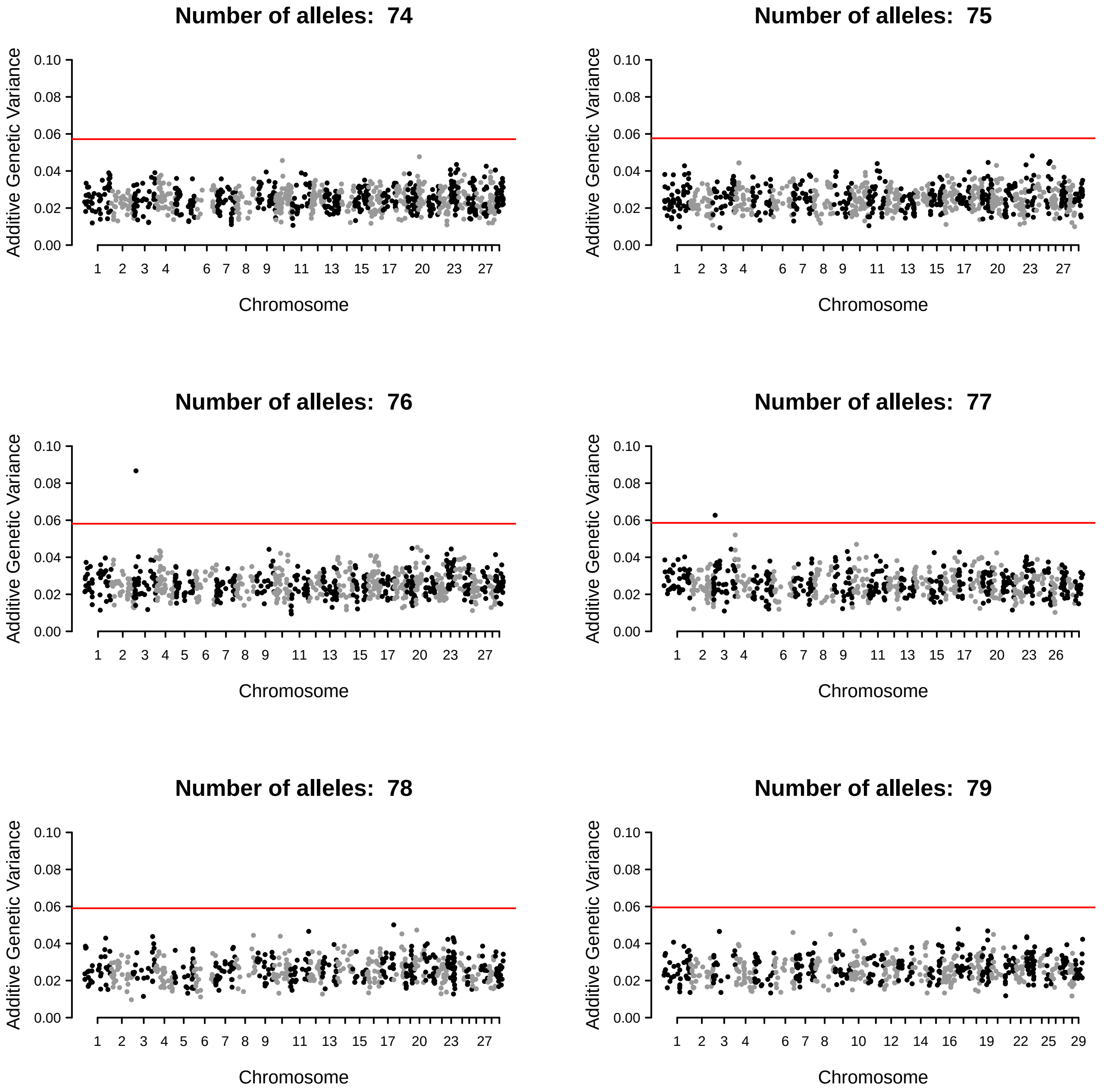

Number of alleles: 74
Additive Genetic Variance
0.00 0.02 0.04 0.06 0.08 0.10
1 2 3 4 6 7 8 9 11 13 15 17 20 23 27
Chromosome
Number of alleles: 75
Additive Genetic Variance
0.00 0.02 0.04 0.06 0.08 0.10
1 2 3 4 6 7 8 9 11 13 15 17 20 23 27
Chromosome
Number of alleles: 76
Additive Genetic Variance
0.00 0.02 0.04 0.06 0.08 0.10
1 2 3 4 5 6 7 8 9 11 13 15 17 20 23 27
Chromosome
Number of alleles: 77
Additive Genetic Variance
0.00 0.02 0.04 0.06 0.08 0.10
1 2 3 4 6 7 8 9 11 13 15 17 20 23 26
Chromosome
Number of alleles: 78
Additive Genetic Variance
0.00 0.02 0.04 0.06 0.08 0.10
1 2 3 4 5 6 7 8 9 11 13 15 17 20 23 27
Chromosome
Number of alleles: 79
Additive Genetic Variance
0.00 0.02 0.04 0.06 0.08 0.10
1 2 3 4 6 7 8 10 12 14 16 19 22 25 29
Chromosome

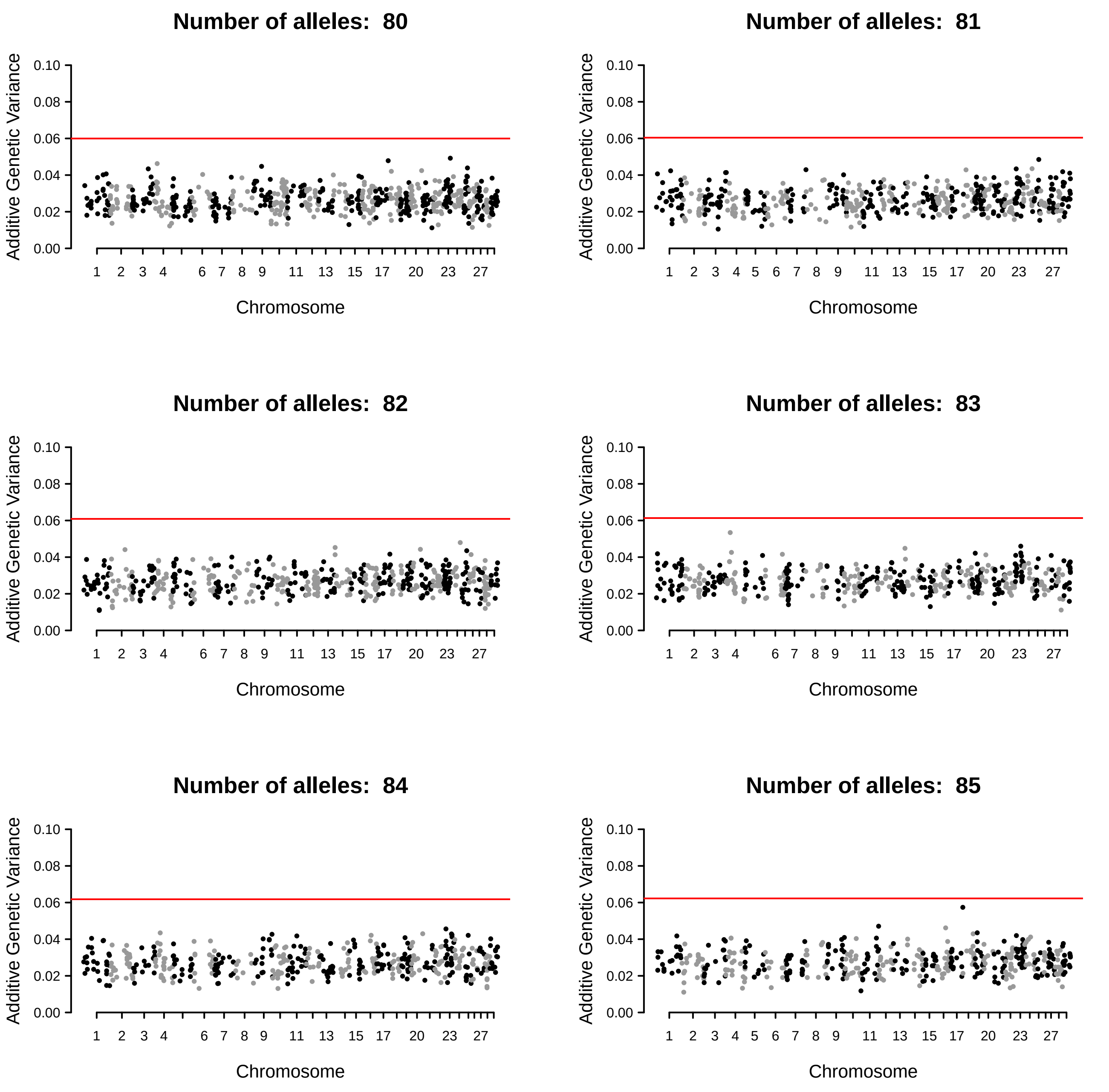

Number of alleles: 80
Number of alleles: 81
Number of alleles: 82
Number of alleles: 83
Number of alleles: 84
Number of alleles: 85
Additive Genetic Variance
0.00
0.02
0.04
0.06
0.08
0.10
Chromosome
1
2
3
4
5
6
7
8
9
11
13
15
17
20
23
27

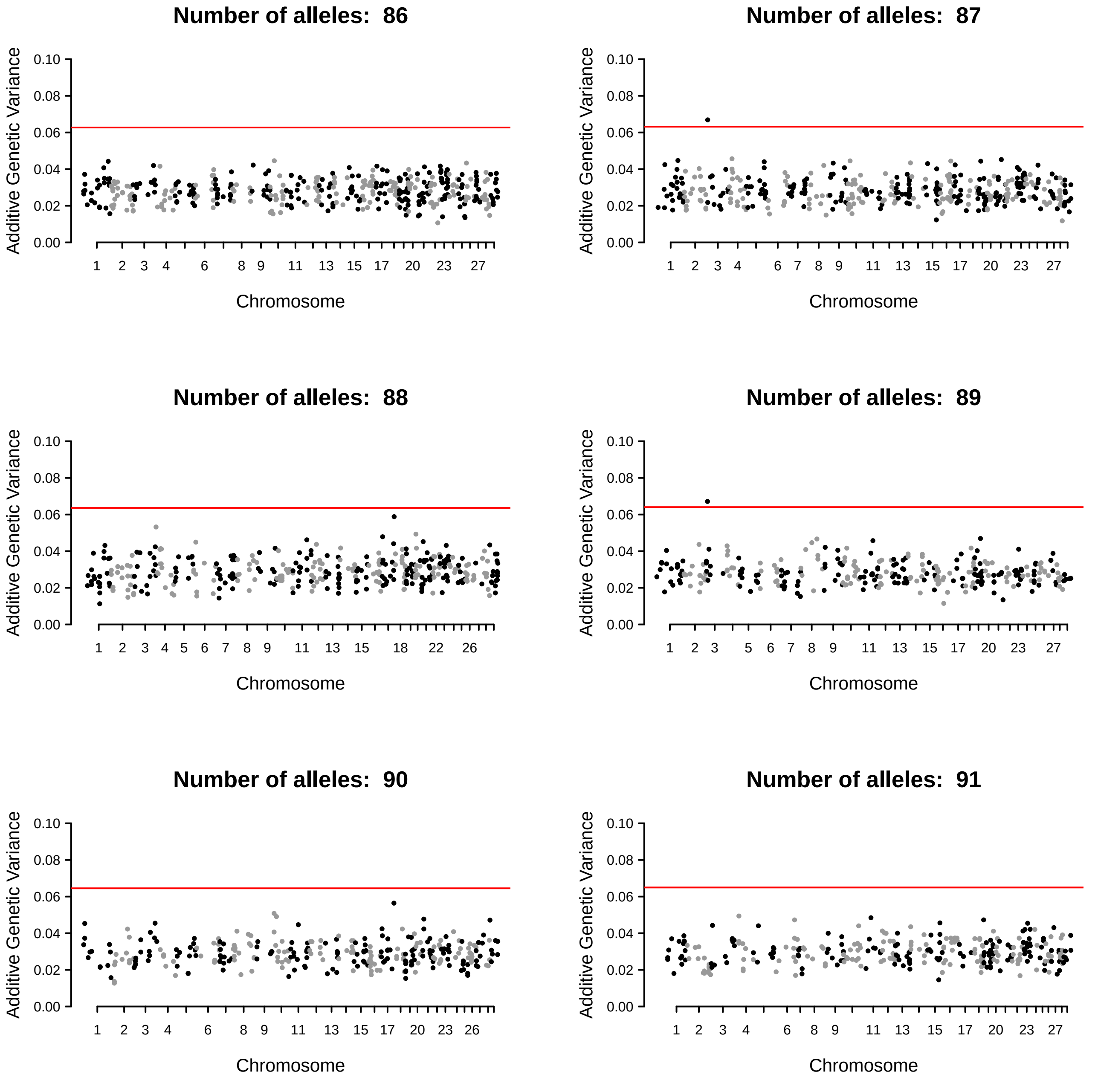
Number of alleles: 86
Number of alleles: 87
Number of alleles: 88
Number of alleles: 89
Number of alleles: 90
Number of alleles: 91
Additive Genetic Variance
0.00 0.02 0.04 0.06 0.08 0.10
Chromosome
1 2 3 4 6 8 9 11 13 15 17 20 23 27
1 2 3 4 6 7 8 9 11 13 15 17 20 23 27
1 2 3 4 5 6 7 8 9 11 13 15 18 22 26
1 2 3 5 6 7 8 9 11 13 15 17 20 23 27
1 2 3 4 6 8 9 11 13 15 17 20 23 26
1 2 3 4 6 8 9 11 13 15 17 20 23 27

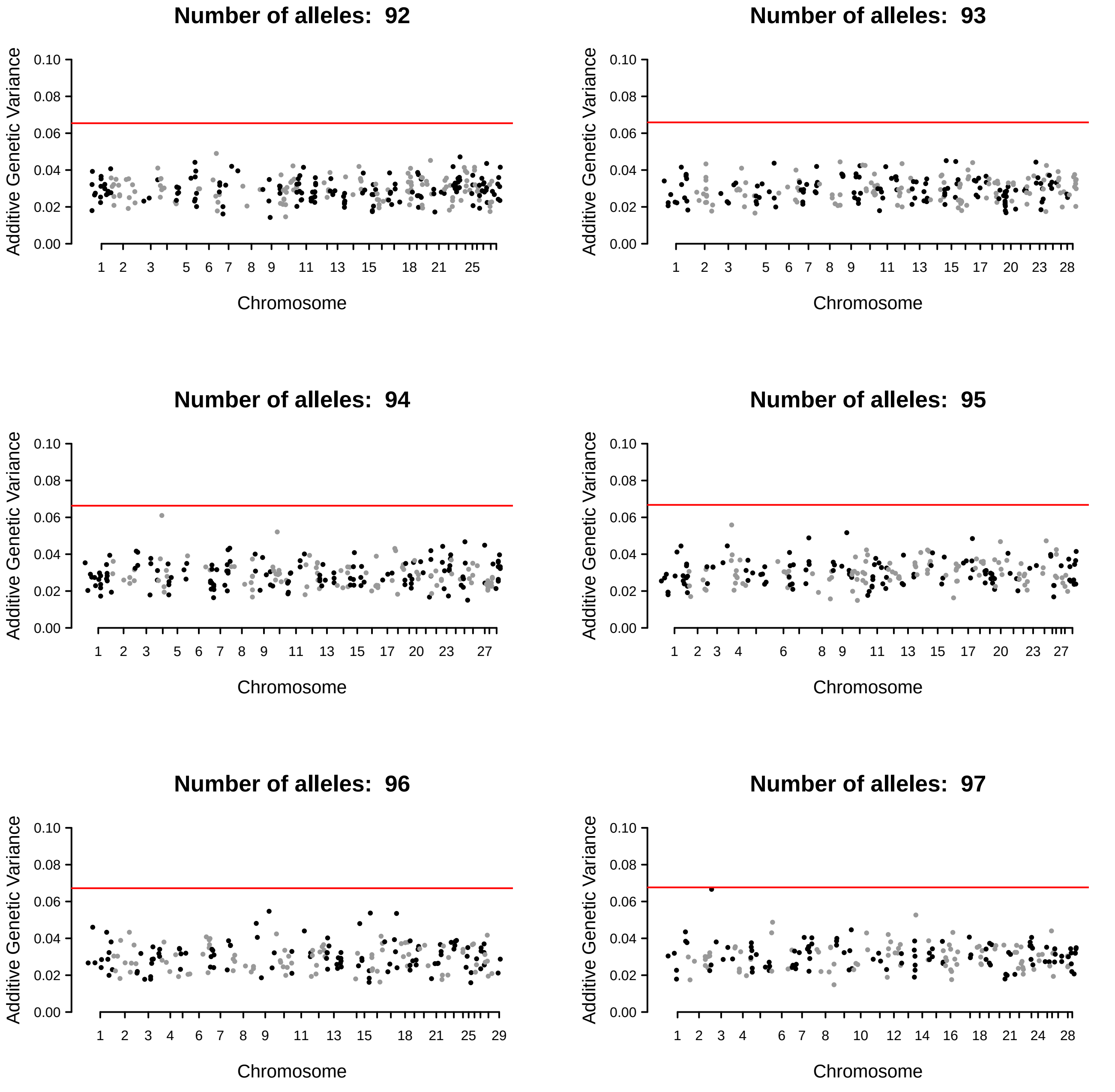
Number of alleles: 92
Number of alleles: 93
Number of alleles: 94
Number of alleles: 95
Number of alleles: 96
Number of alleles: 97
Additive Genetic Variance
0.00 0.02 0.04 0.06 0.08 0.10
Chromosome
1 2 3 5 6 7 8 9 11 13 15 18 21 25
1 2 3 5 6 7 8 9 11 13 15 17 20 23 28
1 2 3 5 6 7 8 9 11 13 15 17 20 23 27
1 2 3 4 6 8 9 11 13 15 17 20 23 27
1 2 3 4 6 7 8 9 11 13 15 18 21 25 29
1 2 3 4 6 7 8 10 12 14 16 18 21 24 28

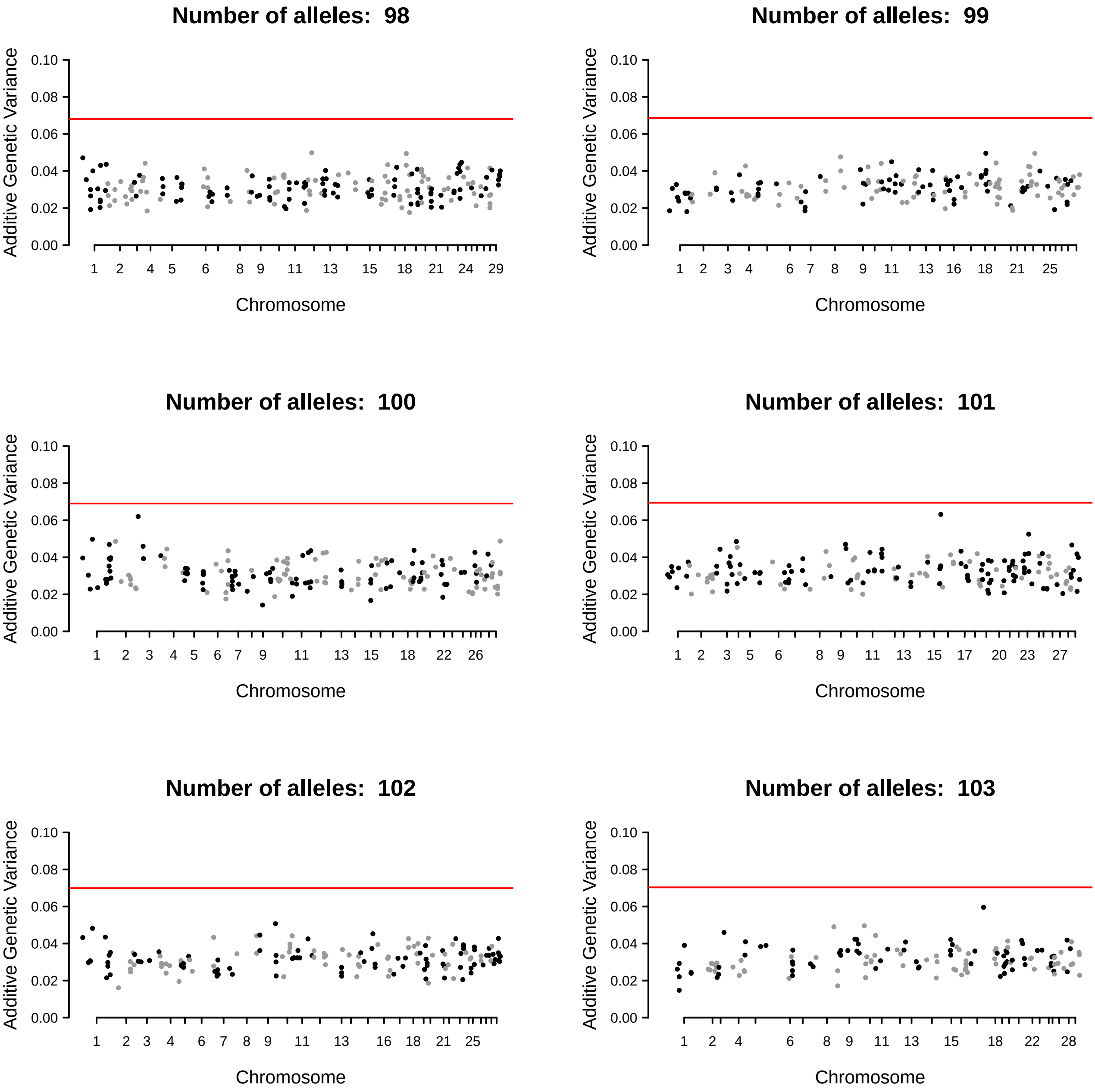
Number of alleles: 98
Number of alleles: 99
Number of alleles: 100
Number of alleles: 101
Number of alleles: 102
Number of alleles: 103
Additive Genetic Variance
0.00
0.02
0.04
0.06
0.08
0.10
Chromosome
1 2 4 5 6 8 9 11 13 15 18 21 24 29
1 2 3 4 6 7 8 9 11 13 16 18 21 25
1 2 3 4 5 6 7 9 11 13 15 18 22 26
1 2 3 5 6 8 9 11 13 15 17 20 23 27
1 2 3 4 6 7 8 9 11 13 16 18 21 25
1 2 4 6 8 9 11 13 15 18 22 28

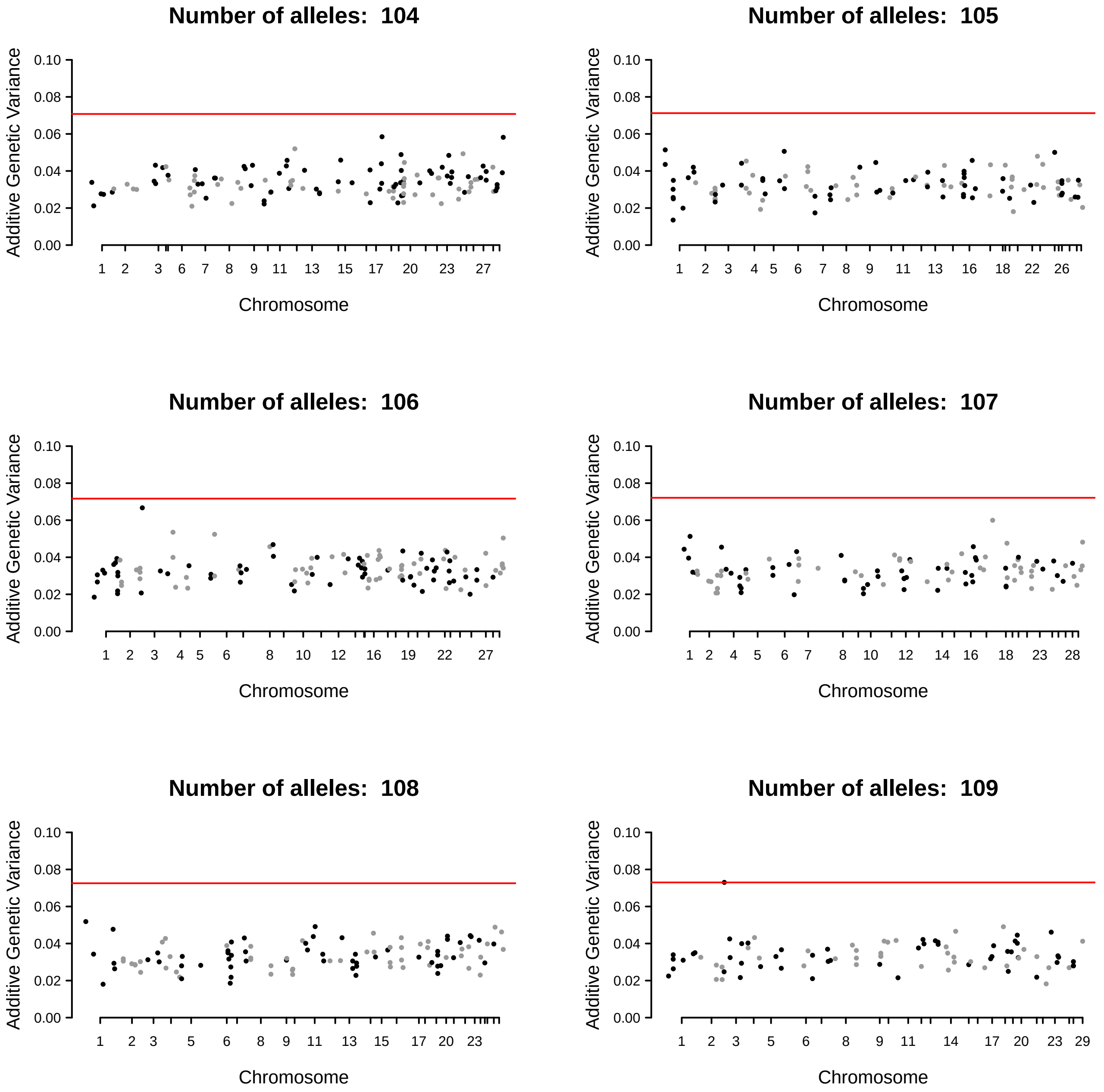

Number of alleles: 104
Number of alleles: 105
Number of alleles: 106
Number of alleles: 107
Number of alleles: 108
Number of alleles: 109
Additive Genetic Variance
0.00
0.02
0.04
0.06
0.08
0.10
Chromosome
1 2 3 6 7 8 9 11 13 15 17 20 23 27
1 2 3 4 5 6 7 8 9 11 13 16 18 22 26
1 2 3 4 5 6 8 10 12 16 19 22 27
1 2 4 5 6 7 8 10 12 14 16 18 23 28
1 2 3 5 6 8 9 11 13 15 17 20 23
1 2 3 5 6 8 9 11 14 17 20 23 29
