# Supplementary material for: Sliding window haplotype approaches overcome single SNP analysis limitations in identifying genes for meat tenderness in Nelore cattle
Source: BMC Genet. 2019 Jan 14;20:8. doi: 10.1186/s12863-019-0713-4 (PMC6332854; doi:10.1186/s12863-019-0713-4)
Supplement: Supplementary file 4 — QQ plots for haplotype-based association analyses for each number of alleles at haplotyped loci. (PDF 2144 kb) [file 12863_2019_713_MOESM4_ESM.pdf]

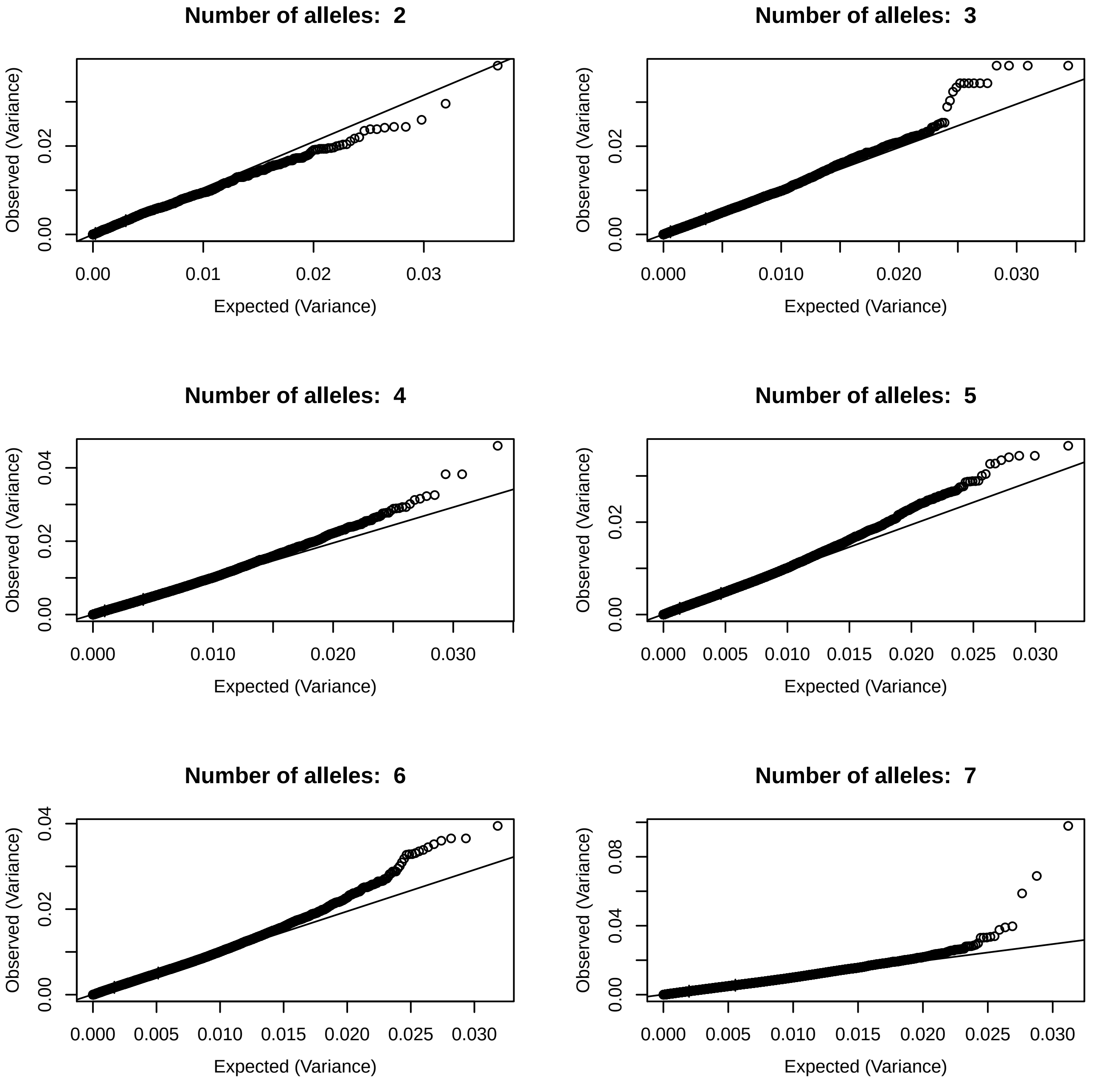
Number of alleles: 2
Number of alleles: 3
Number of alleles: 4
Number of alleles: 5
Number of alleles: 6
Number of alleles: 7
Observed (Variance)
Expected (Variance)
0.00
0.02
0.04
0.08
0.01
0.03
0.000
0.005
0.010
0.015
0.020
0.025
0.030

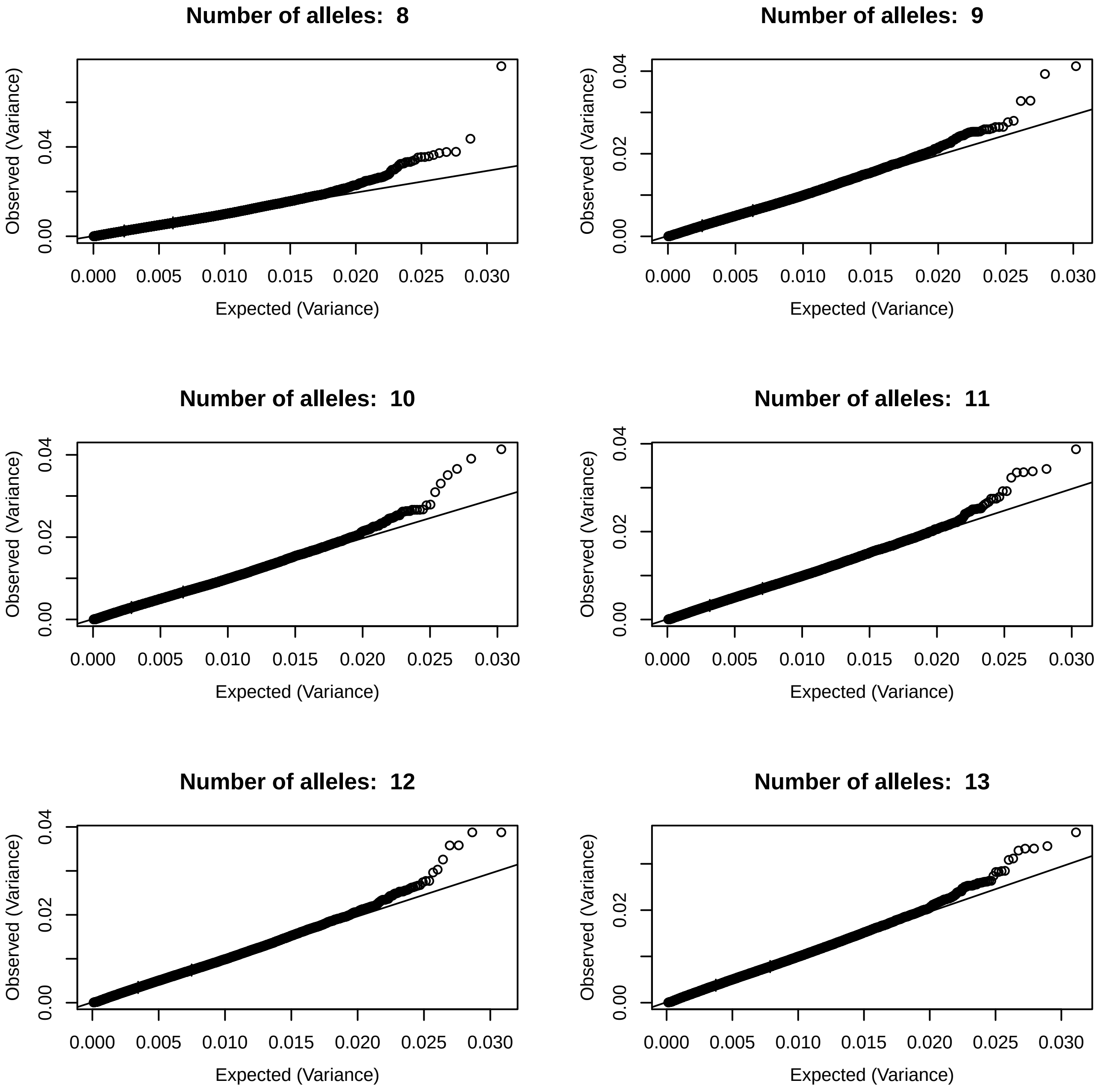
Number of alleles: 8
Number of alleles: 9
Number of alleles: 10
Number of alleles: 11
Number of alleles: 12
Number of alleles: 13
Observed (Variance)
Expected (Variance)
0.00
0.02
0.04
0.000
0.005
0.010
0.015
0.020
0.025
0.030

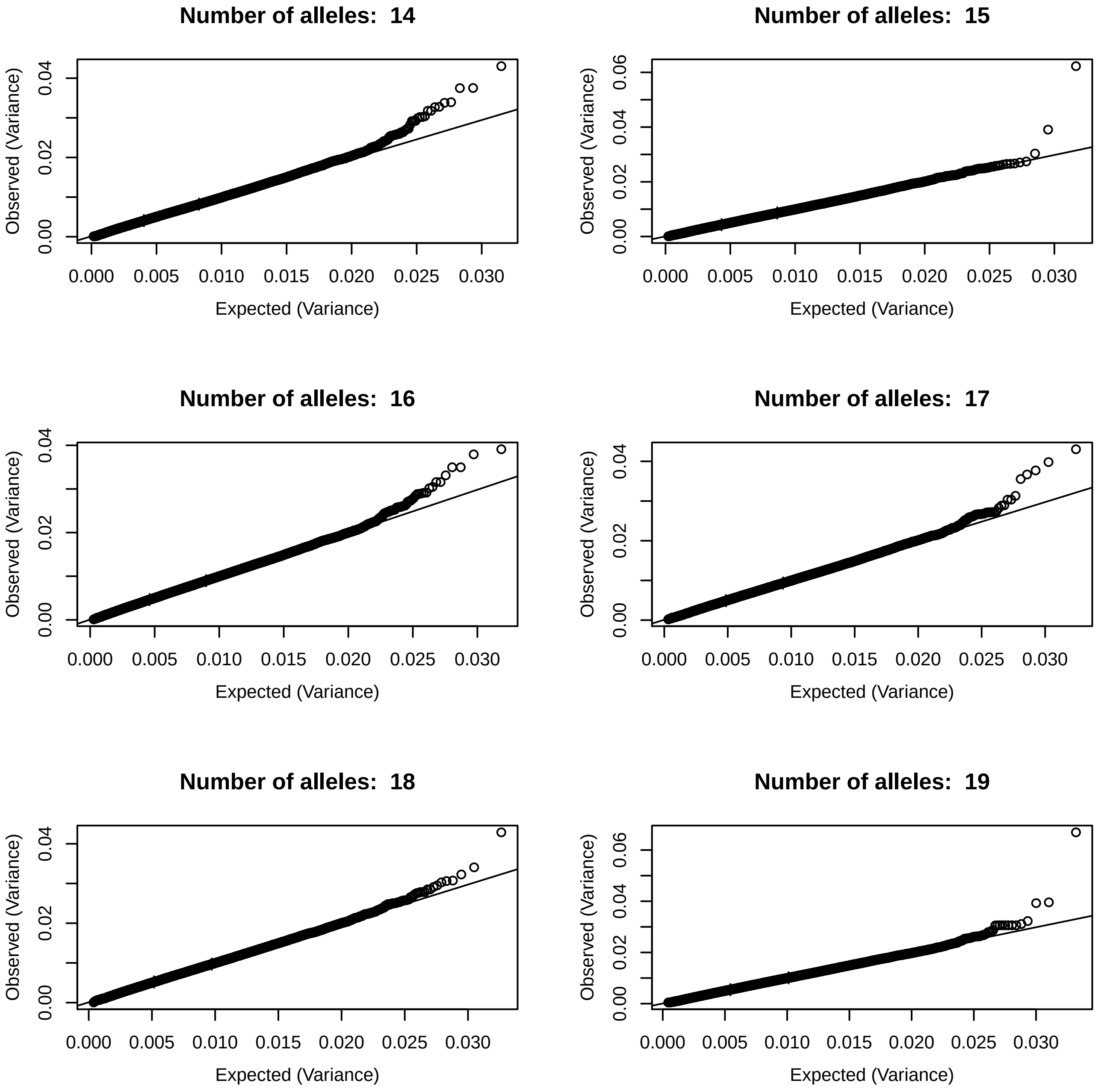
Number of alleles: 14
Number of alleles: 15
Number of alleles: 16
Number of alleles: 17
Number of alleles: 18
Number of alleles: 19
Observed (Variance)
Expected (Variance)
0.00
0.02
0.04
0.06
0.000
0.005
0.010
0.015
0.020
0.025
0.030

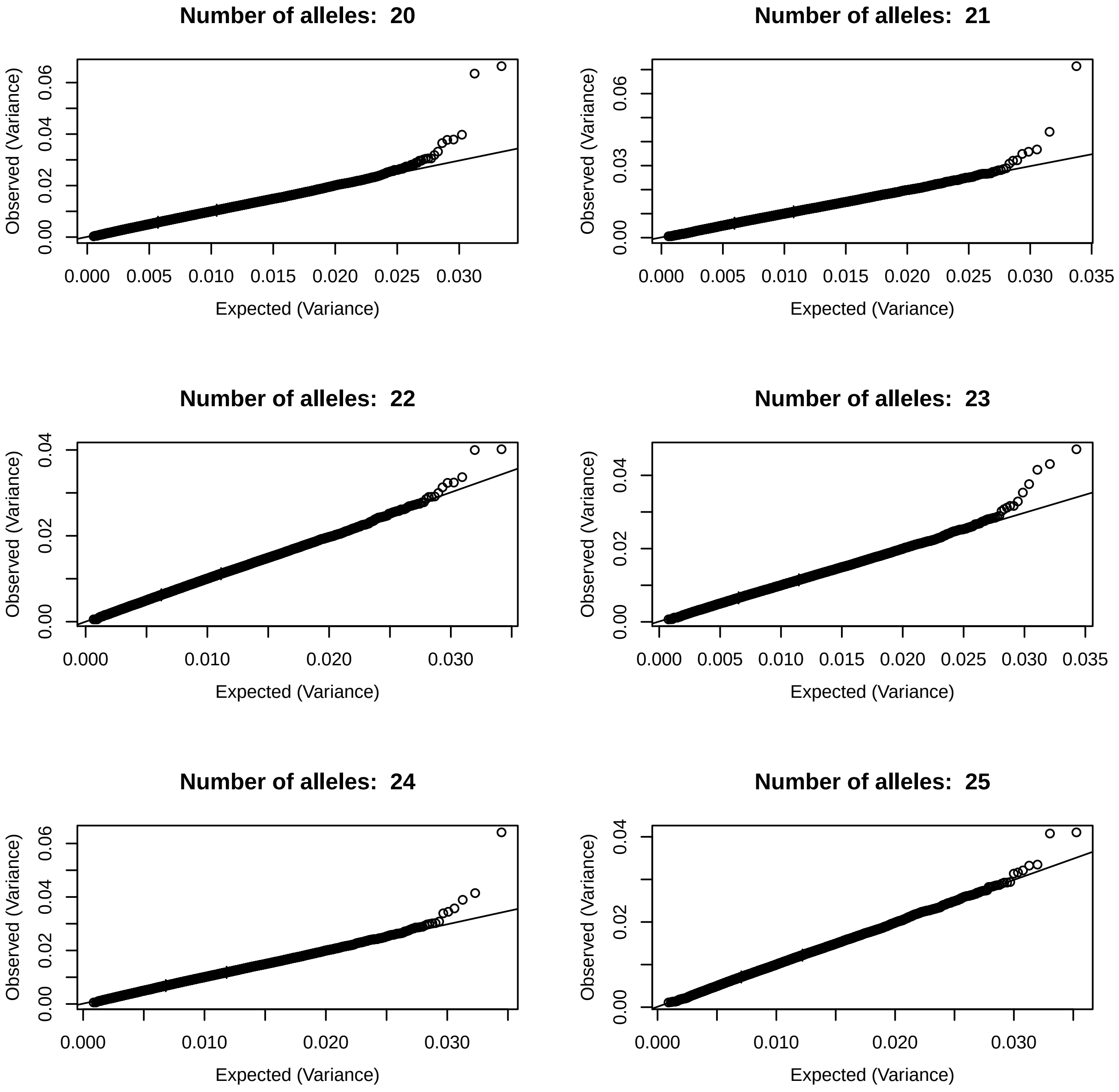
Number of alleles: 20
Number of alleles: 21
Number of alleles: 22
Number of alleles: 23
Number of alleles: 24
Number of alleles: 25
Observed (Variance)
Expected (Variance)
0.00
0.02
0.03
0.04
0.06
0.000
0.005
0.010
0.015
0.020
0.025
0.030
0.035

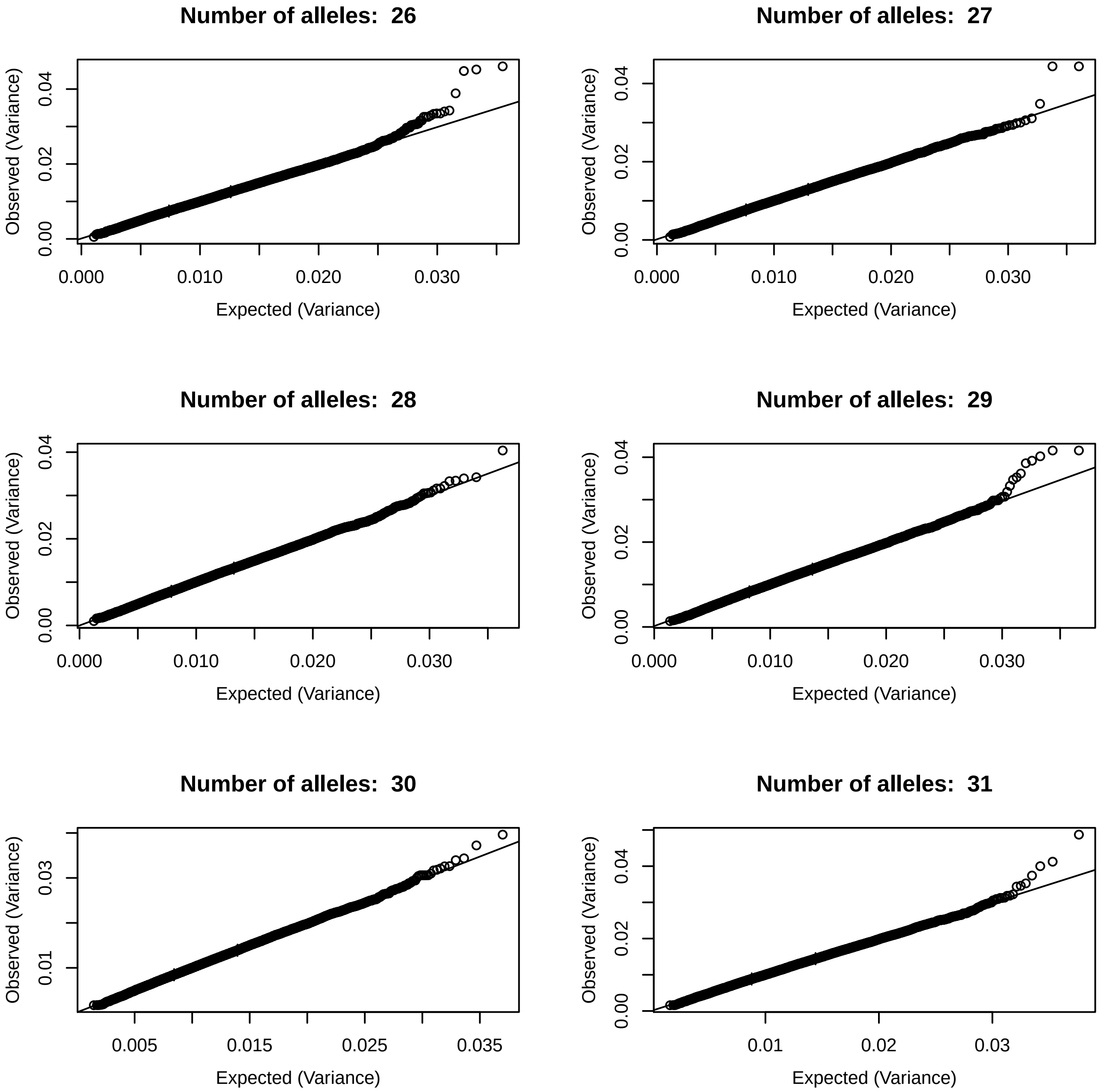
Number of alleles: 26
Number of alleles: 27
Number of alleles: 28
Number of alleles: 29
Number of alleles: 30
Number of alleles: 31
Observed (Variance)
Expected (Variance)
0.00
0.02
0.04
0.01
0.03
0.000
0.010
0.020
0.030
0.005
0.015
0.025
0.035

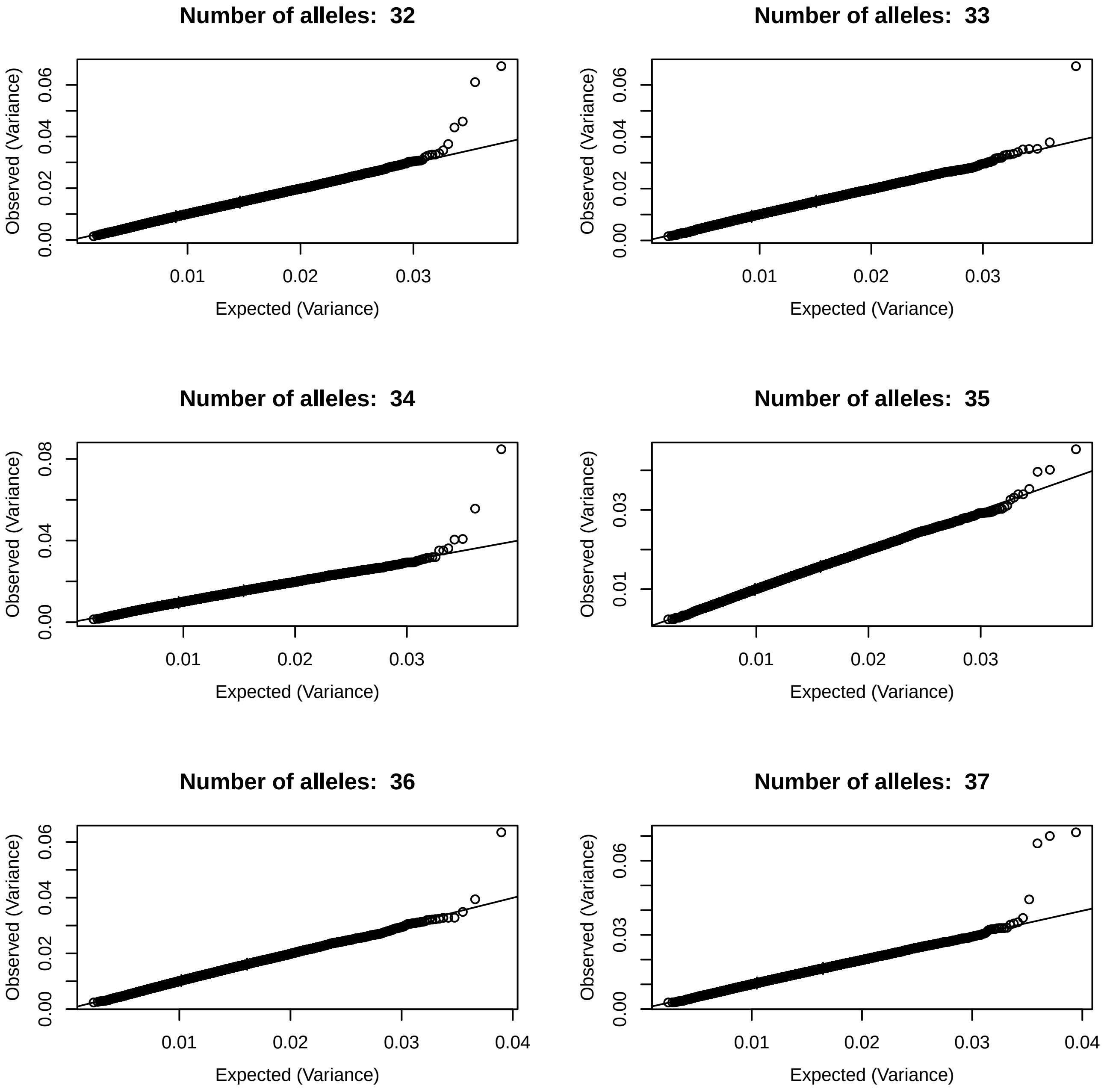
Number of alleles: 32
Number of alleles: 33
Number of alleles: 34
Number of alleles: 35
Number of alleles: 36
Number of alleles: 37
Observed (Variance)
Expected (Variance)
0.00
0.01
0.02
0.03
0.04
0.06
0.08

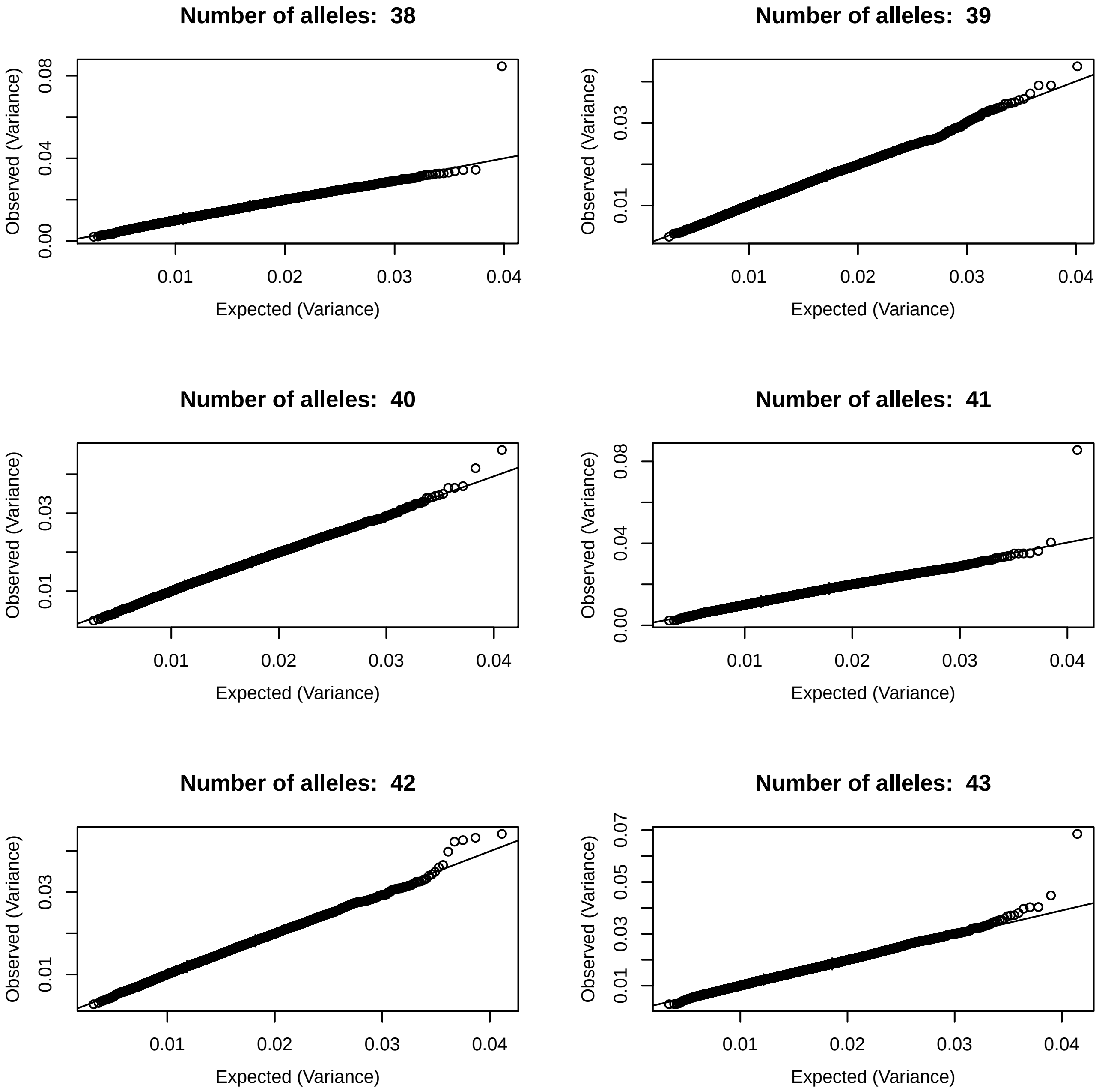
Number of alleles: 38
Number of alleles: 39
Number of alleles: 40
Number of alleles: 41
Number of alleles: 42
Number of alleles: 43
Observed (Variance)
Expected (Variance)
0.00
0.04
0.08
0.01
0.03
0.05
0.07
0.01
0.02
0.03
0.04

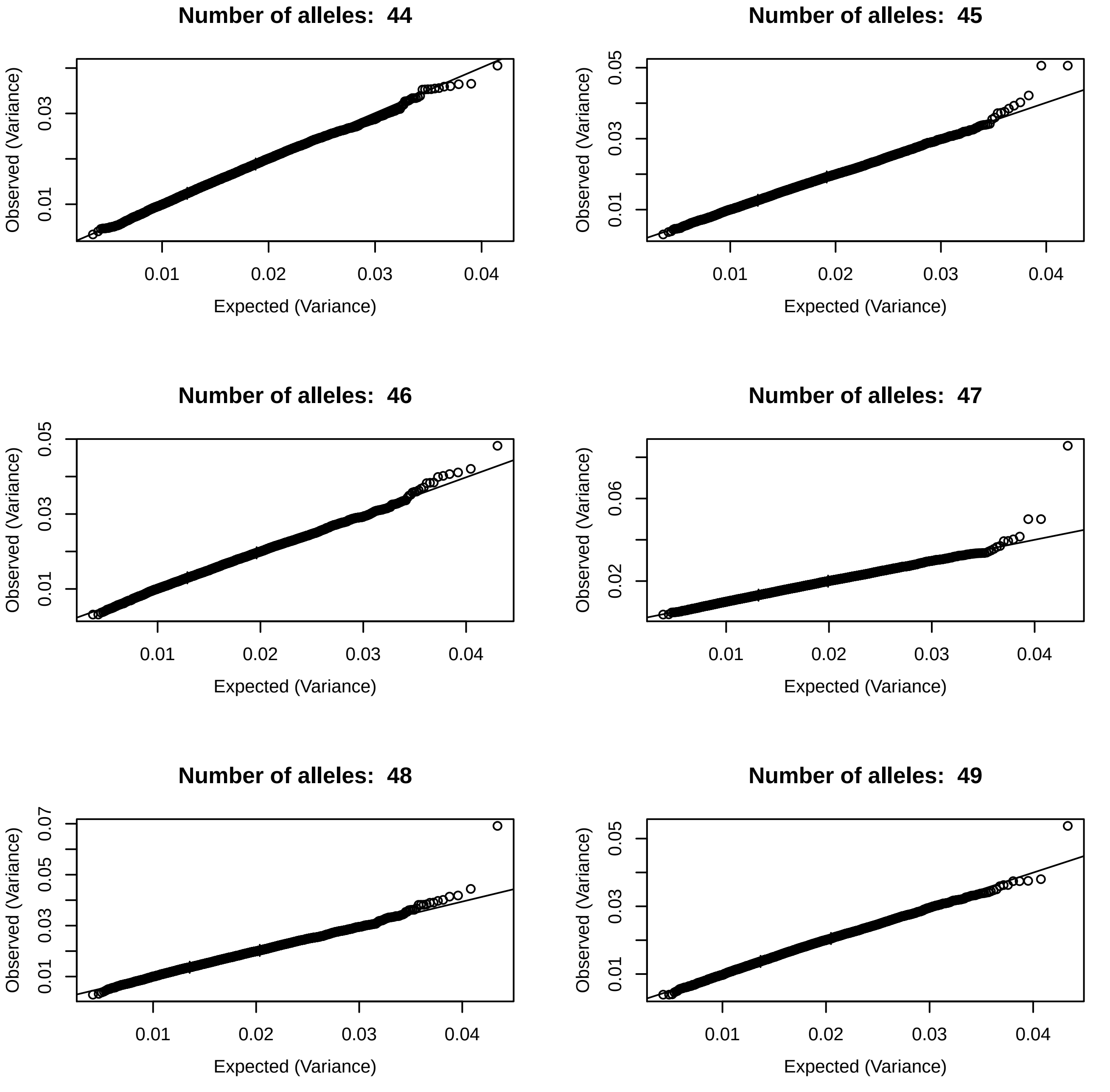
Number of alleles: 44
Number of alleles: 45
Number of alleles: 46
Number of alleles: 47
Number of alleles: 48
Number of alleles: 49
Observed (Variance)
Expected (Variance)
0.01
0.02
0.03
0.04
0.05
0.06
0.07

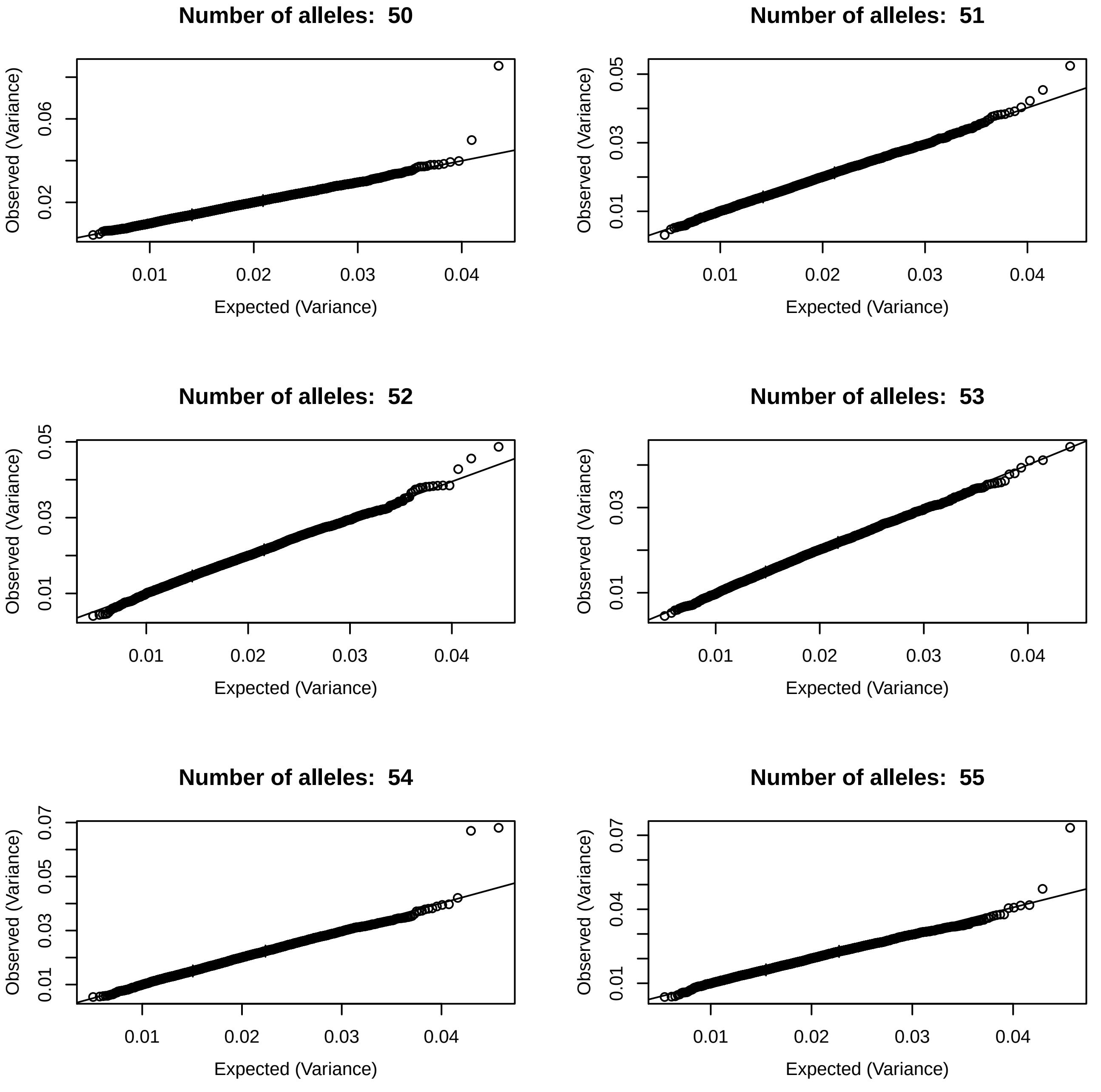
Number of alleles: 50
Number of alleles: 51
Number of alleles: 52
Number of alleles: 53
Number of alleles: 54
Number of alleles: 55
Observed (Variance)
Expected (Variance)
0.01
0.02
0.03
0.04
0.05
0.06
0.07

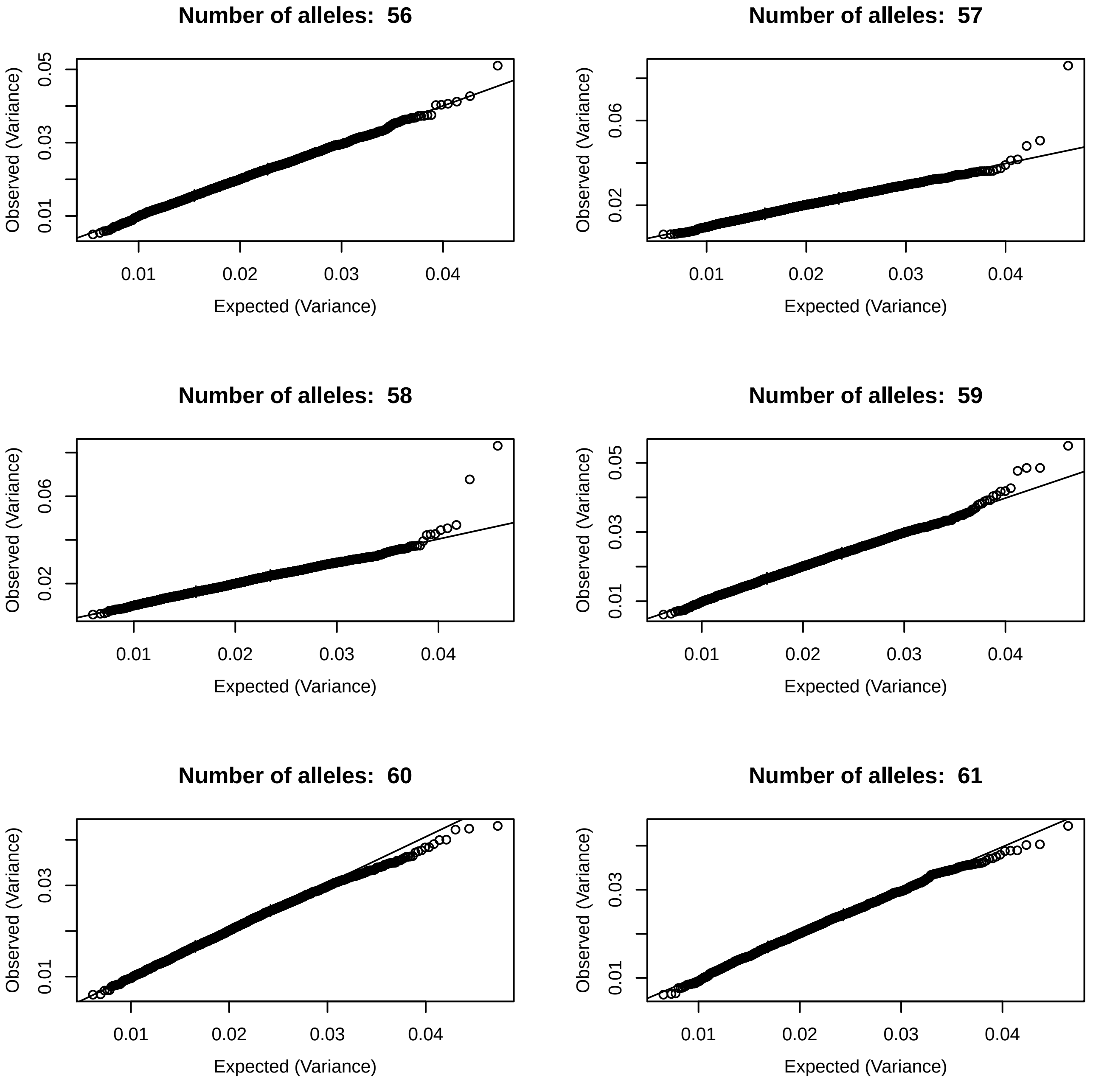

Number of alleles: 56
Number of alleles: 57
Number of alleles: 58
Number of alleles: 59
Number of alleles: 60
Number of alleles: 61
Observed (Variance)
Expected (Variance)
0.01
0.02
0.03
0.04
0.05
0.06

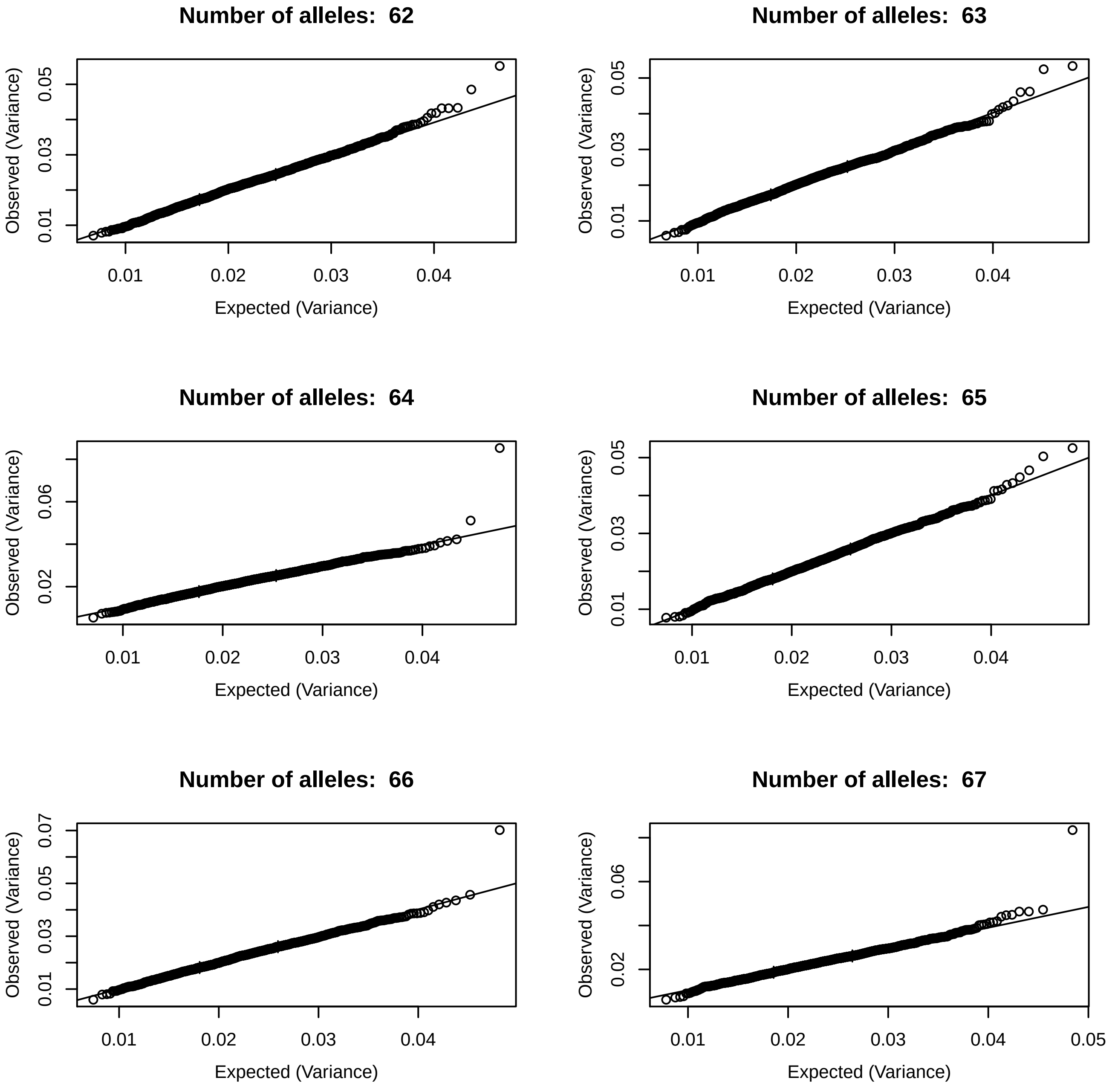
Number of alleles: 62
Observed (Variance)
Expected (Variance)
Number of alleles: 63
Observed (Variance)
Expected (Variance)
Number of alleles: 64
Observed (Variance)
Expected (Variance)
Number of alleles: 65
Observed (Variance)
Expected (Variance)
Number of alleles: 66
Observed (Variance)
Expected (Variance)
Number of alleles: 67
Observed (Variance)
Expected (Variance)

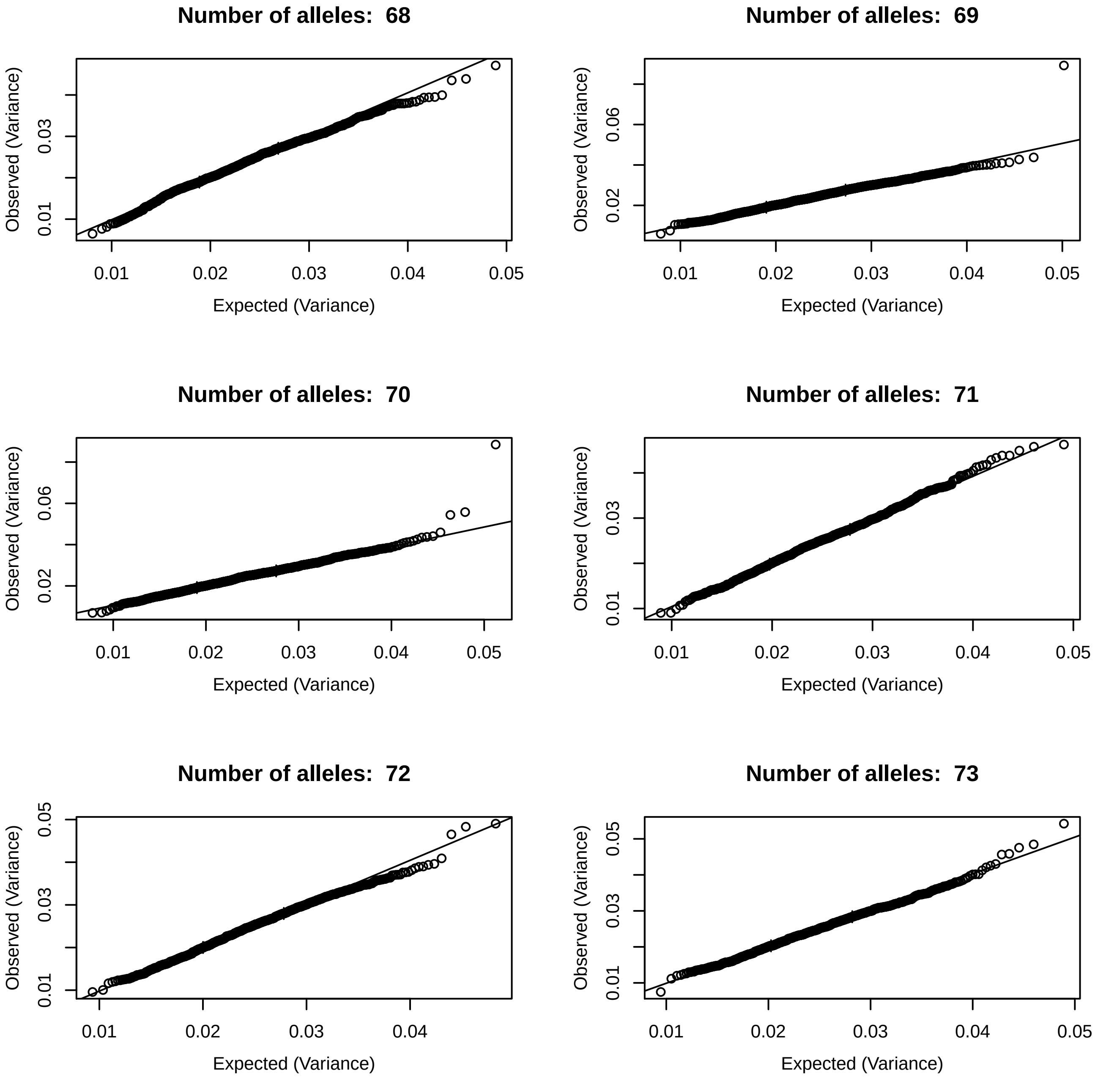

Number of alleles: 68
Number of alleles: 69
Number of alleles: 70
Number of alleles: 71
Number of alleles: 72
Number of alleles: 73
Observed (Variance)
Expected (Variance)
0.01
0.02
0.03
0.04
0.05
0.06

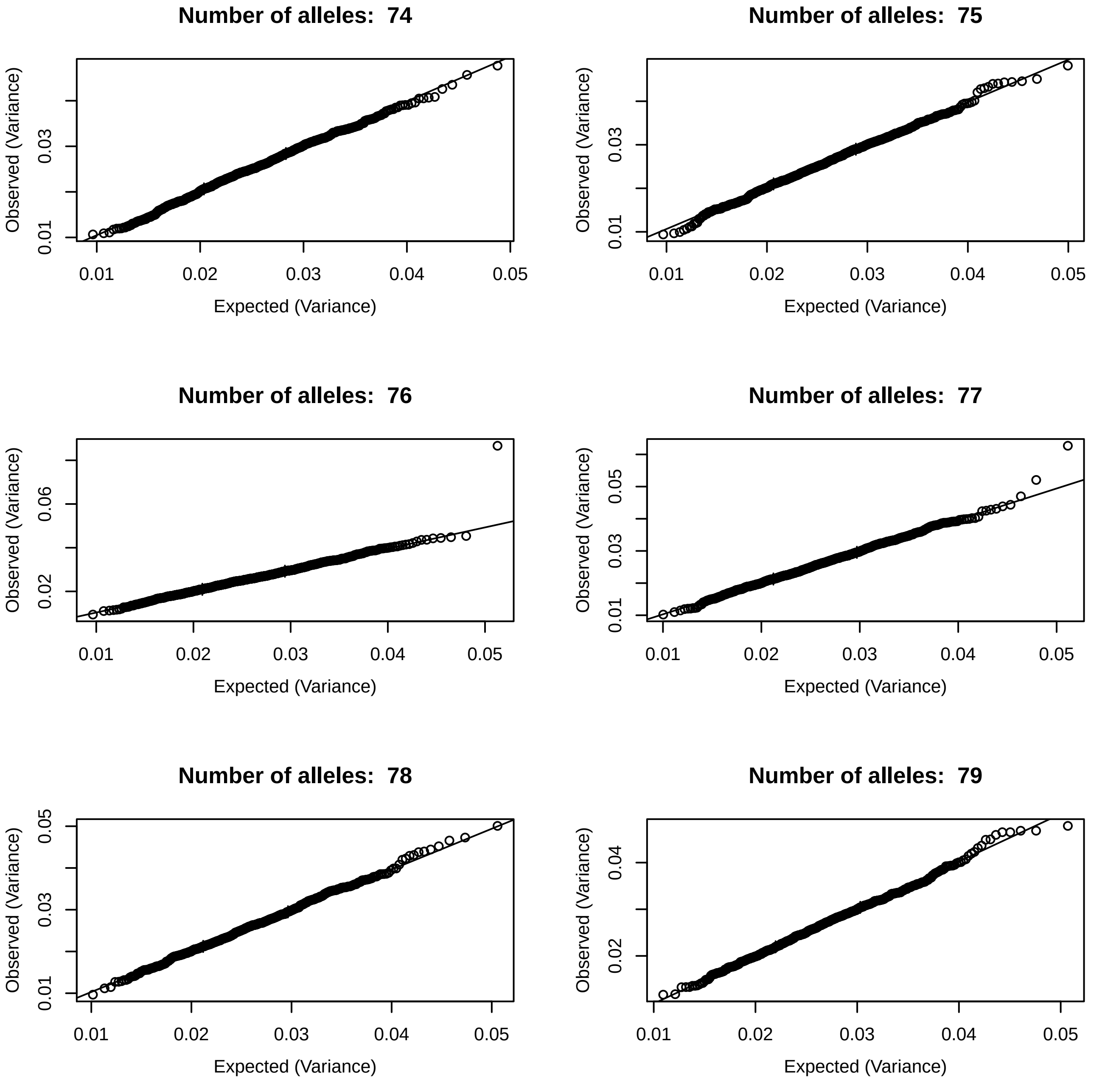
Number of alleles: 74
Number of alleles: 75
Number of alleles: 76
Number of alleles: 77
Number of alleles: 78
Number of alleles: 79
Observed (Variance)
Expected (Variance)
0.01
0.02
0.03
0.04
0.05
0.06

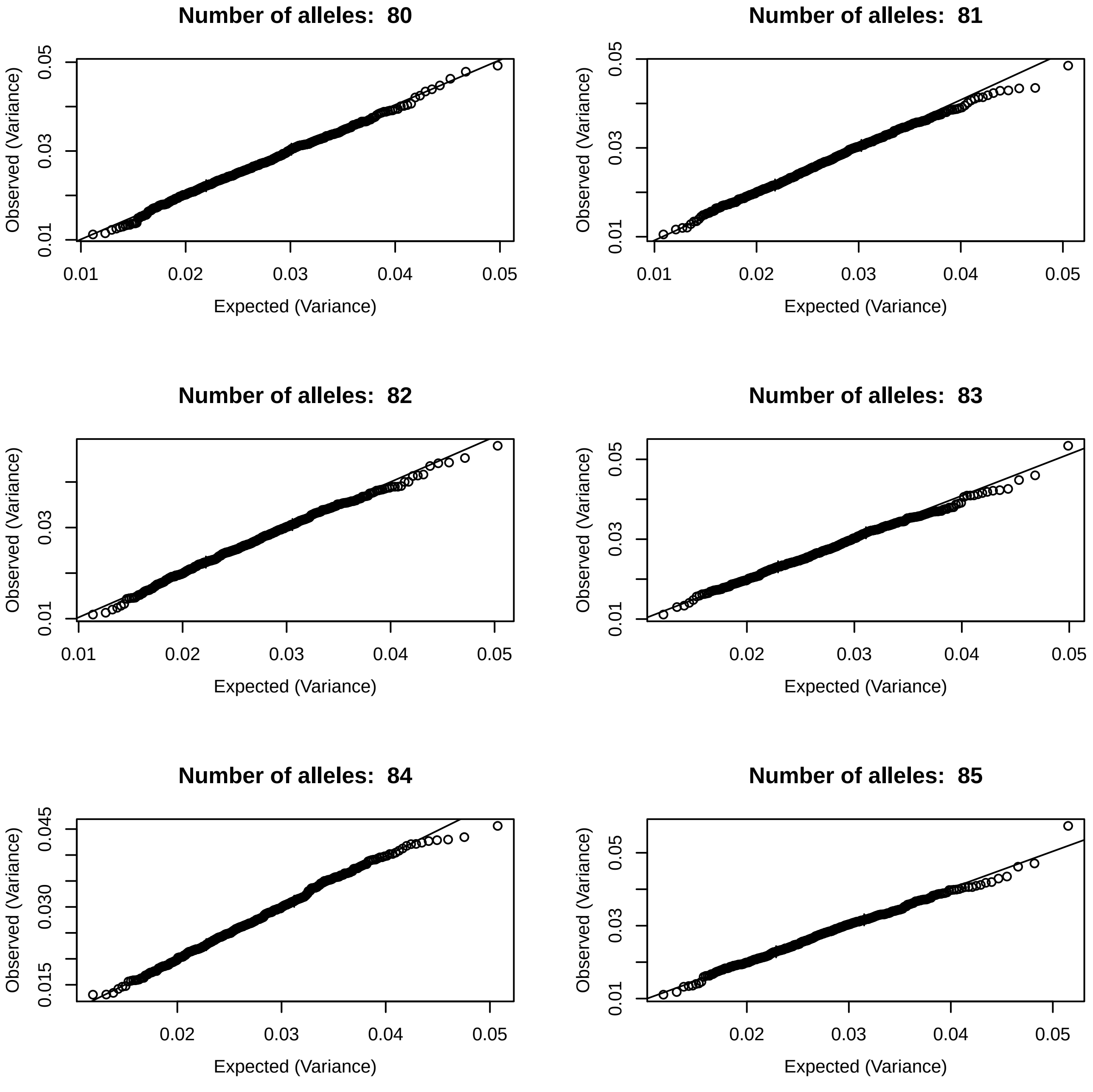

Number of alleles: 80
Number of alleles: 81
Number of alleles: 82
Number of alleles: 83
Number of alleles: 84
Number of alleles: 85
Observed (Variance)
Expected (Variance)
0.01
0.02
0.03
0.04
0.05
0.015
0.030
0.045

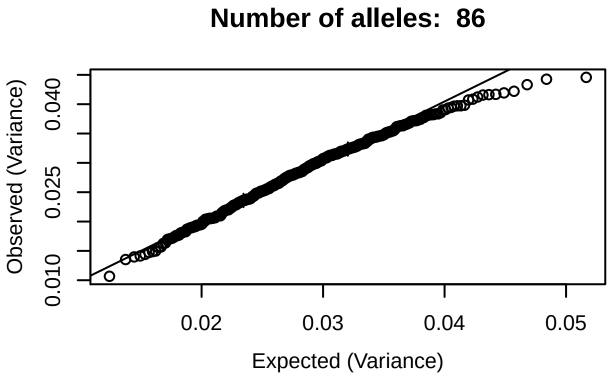
Number of alleles: 86
Observed (Variance)
0.010
0.025
0.040
0.02
0.03
0.04
0.05
Expected (Variance)

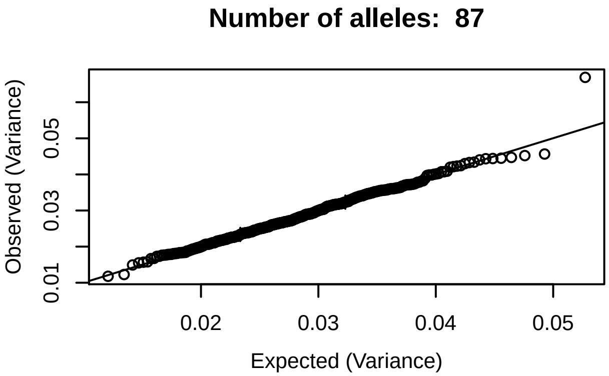
Number of alleles: 87
Observed (Variance)
0.01
0.03
0.05
0.02
0.03
0.04
0.05
Expected (Variance)

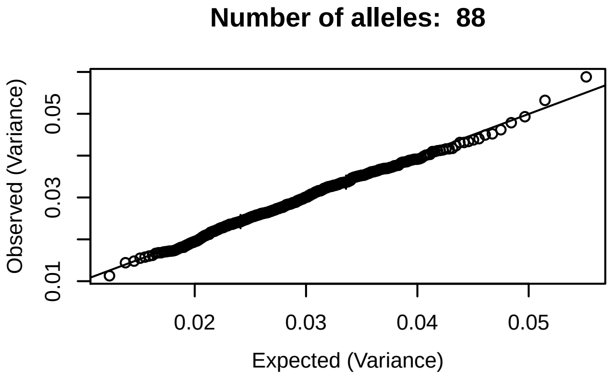
Number of alleles: 88
Observed (Variance)
0.01
0.03
0.05
0.02
0.03
0.04
0.05
Expected (Variance)

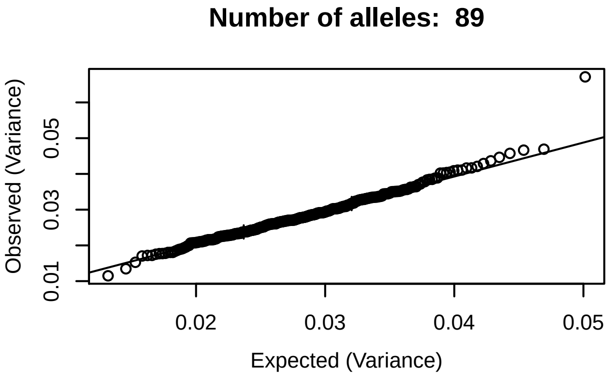
Number of alleles: 89
Observed (Variance)
0.01
0.03
0.05
0.02
0.03
0.04
0.05
Expected (Variance)

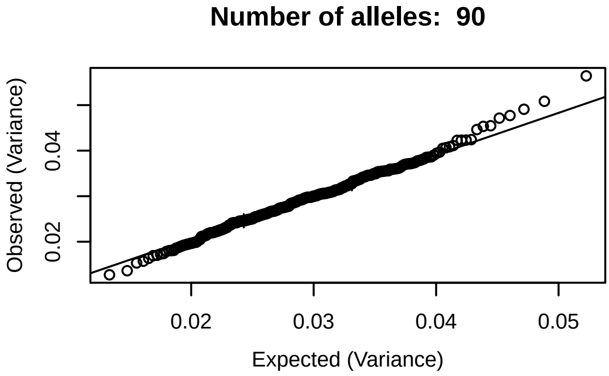
Number of alleles: 90
Observed (Variance)
0.02
0.04
0.02
0.03
0.04
0.05
Expected (Variance)

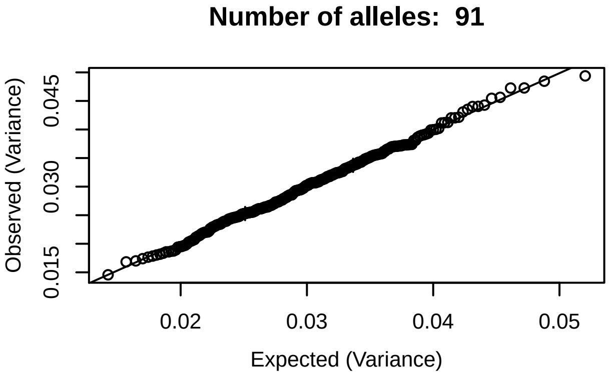
Number of alleles: 91
Observed (Variance)
0.015
0.030
0.045
0.02
0.03
0.04
0.05
Expected (Variance)

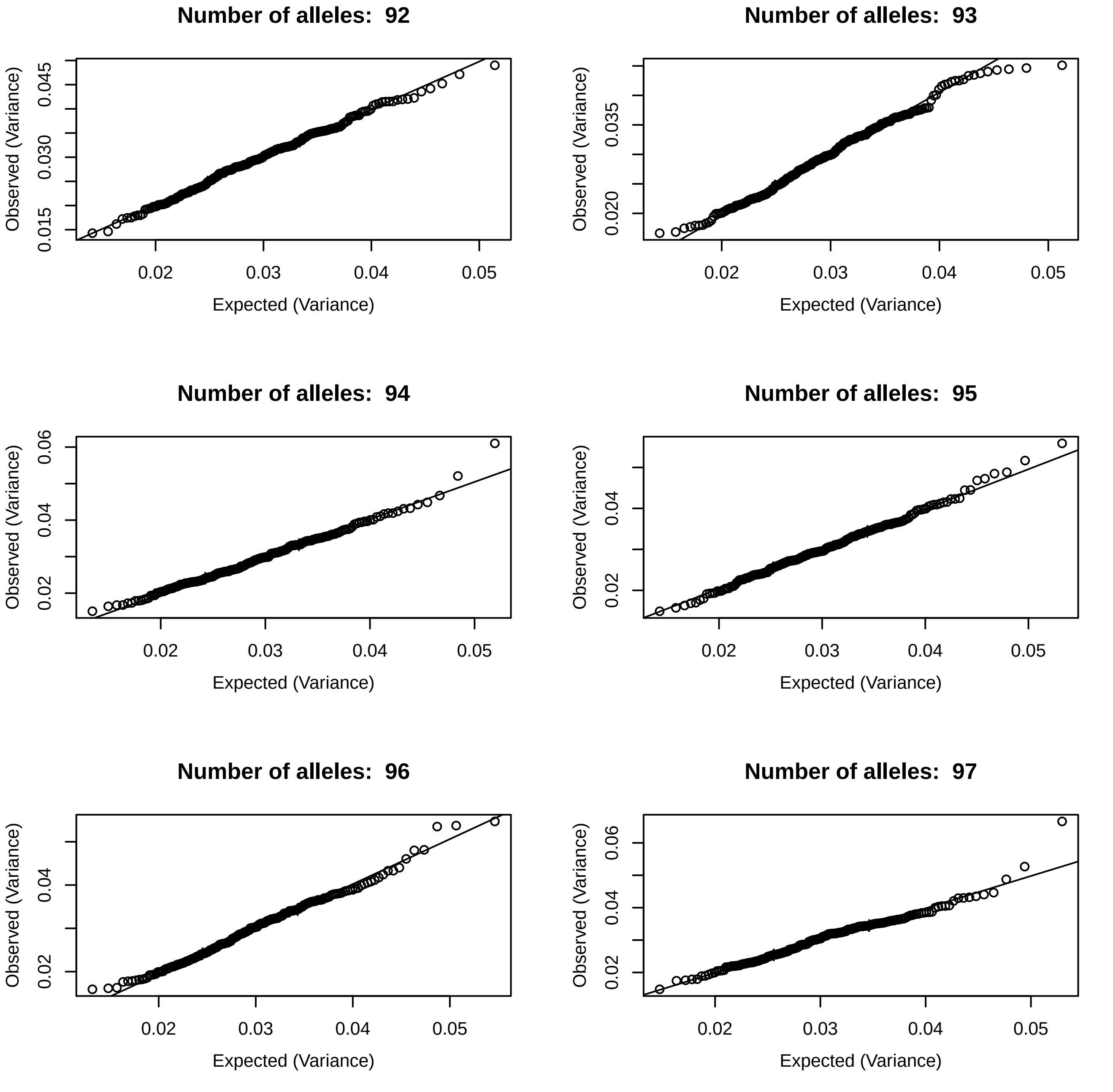
Number of alleles: 92
Observed (Variance)
0.015
0.030
0.045
0.02
0.03
0.04
0.05
Expected (Variance)
Number of alleles: 93
Observed (Variance)
0.020
0.035
0.02
0.03
0.04
0.05
Expected (Variance)
Number of alleles: 94
Observed (Variance)
0.02
0.04
0.06
0.02
0.03
0.04
0.05
Expected (Variance)
Number of alleles: 95
Observed (Variance)
0.02
0.04
0.02
0.03
0.04
0.05
Expected (Variance)
Number of alleles: 96
Observed (Variance)
0.02
0.04
0.02
0.03
0.04
0.05
Expected (Variance)
Number of alleles: 97
Observed (Variance)
0.02
0.04
0.06
0.02
0.03
0.04
0.05
Expected (Variance)

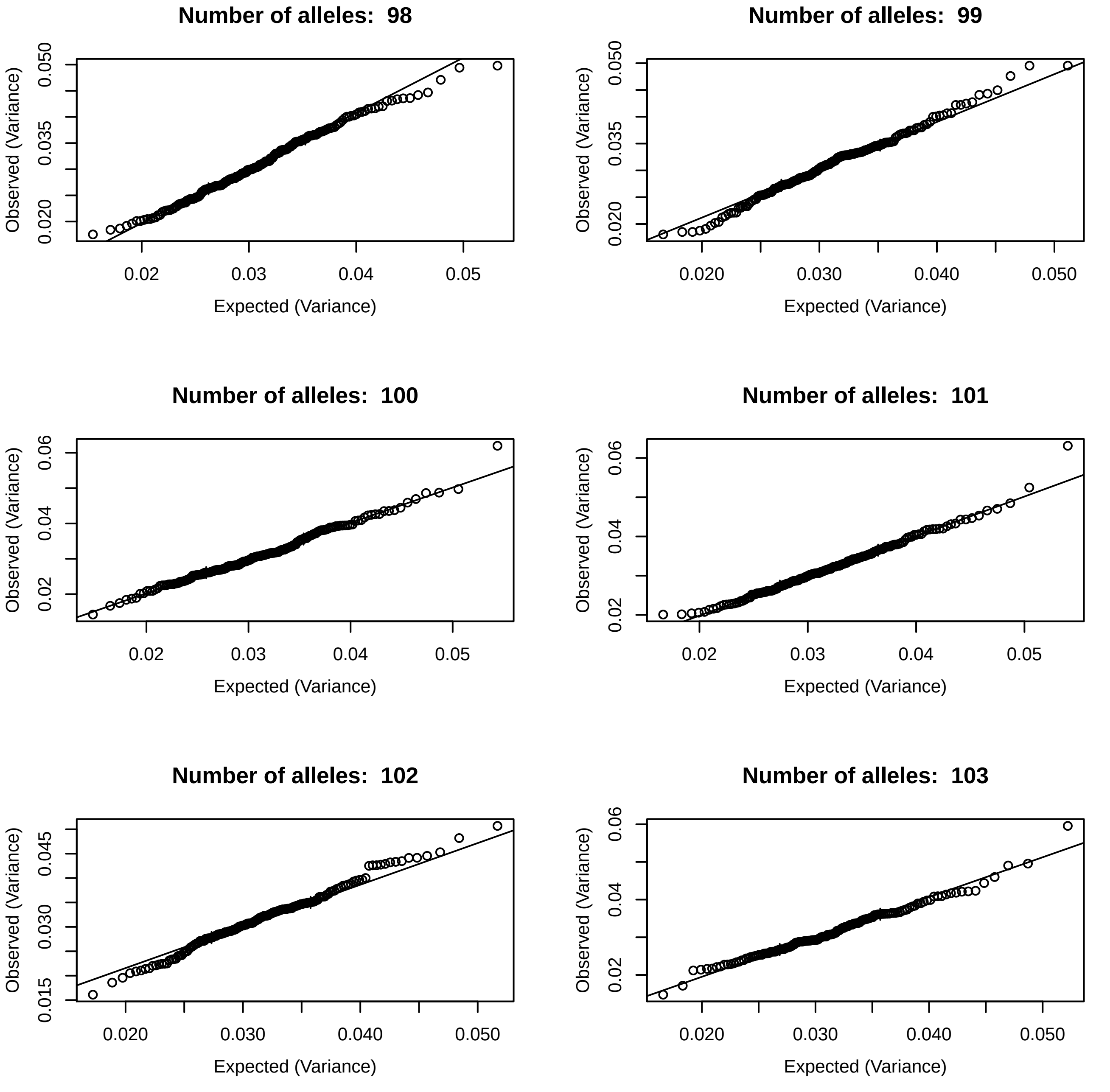
Number of alleles: 98
Number of alleles: 99
Number of alleles: 100
Number of alleles: 101
Number of alleles: 102
Number of alleles: 103
Observed (Variance)
Expected (Variance)

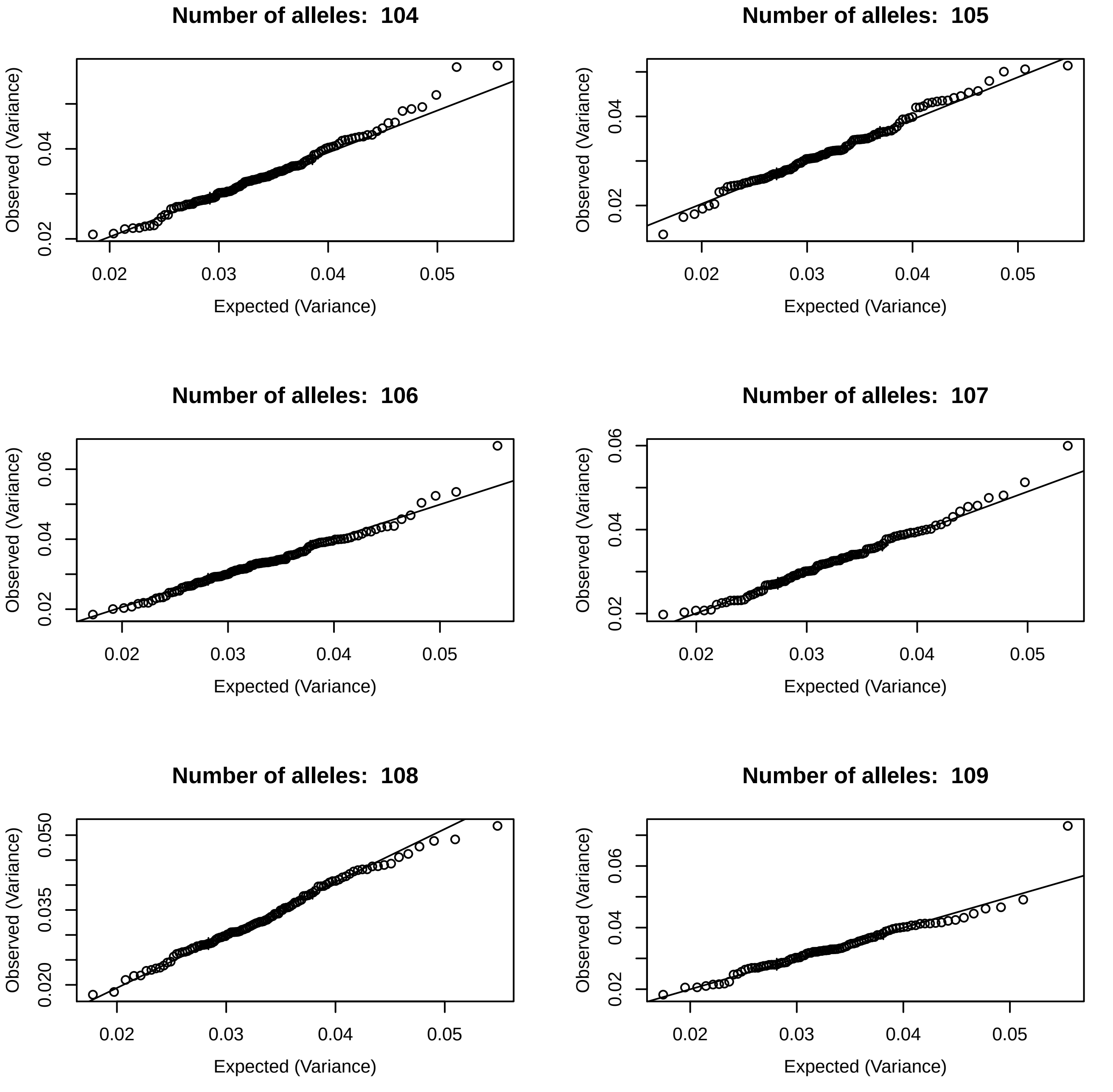
Number of alleles: 104
Number of alleles: 105
Number of alleles: 106
Number of alleles: 107
Number of alleles: 108
Number of alleles: 109
Observed (Variance)
Expected (Variance)
0.02
0.03
0.04
0.05
0.06
0.020
0.035
0.050
